# Tumour‐targeted fluorescence‐guided surgery in gastrointestinal cancer: A systematic review of preclinical and clinical research

**DOI:** 10.1002/ctm2.70615

**Published:** 2026-03-22

**Authors:** Aaya Darai, Evie H. M. Graus, Femke J. A. van der Stroom, Mark Rijpkema, Alexander Vahrmeijer, Denise E. Hilling, Johannes H. W. De Wilt, Merlijn Hutteman

**Affiliations:** ^1^ Department of Surgery Radboud University Medical Centre Nijmegen The Netherlands; ^2^ Department of Medical imaging Nuclear medicine Radboud University Medical Centre Nijmegen The Netherlands; ^3^ Department of Surgery Leiden University Medical Centre Leiden The Netherlands; ^4^ Department of Surgical Oncology and Gastrointestinal Surgery Erasmus MC Cancer Institute University Medical Centre Rotterdam Rotterdam The Netherlands

**Keywords:** gastrointestinal cancers, tumour‐targeted fluorescence‐guided surgery

## Abstract

**Background:**

Gastrointestinal cancers remain a leading cause of cancer‐related morbidity and mortality worldwide, with surgery being central to curative treatment. Tumour‐targeted fluorescence‐guided surgery (tFGS) has emerged as a promising approach to improve intraoperative visualisation and oncological precision.

**Methods:**

We conducted a systematic review of preclinical and clinical studies on tFGS in gastrointestinal oncology, registered in PROSPERO (ID: 558994) and following Preferred Reporting Items for Systematic Reviews and Meta‐Analyses guidelines. Searches of PubMed and Embase identified 133 eligible studies.

**Results:**

Nine tracers have currently been evaluated in clinical trials, targeting the following biomarkers: carcinoembryonic antigen, vascular endothelial growth factor, epidermal growth factor receptor, folate receptor α and/or β, integrin αvβ3, 5‐aminolevulinic acid and acidic tumour microenvironment.

Clinical trials demonstrated that tracers like SGM‐101 and panitumumab–IRDye800CW can achieve high tumour‐to‐background ratios (TBRs) up to 6.1 ex vivo and alter surgical strategy in up to 35% of cases. Preclinical research identified additional promising targets, including mucins, epithelial cell adhesion molecules, urokinase receptors, tumour‐associated glycoproteins, gamma‐glutamyl transferase, organic anion transporting polypeptides, human epidermal growth factors 1/2 and Lewis antibodies, with TBRs frequently exceeding three.

Despite encouraging feasibility and safety data, translation into routine practice is hampered. Confirmation from early health technology assessments is needed to advance tFGS. Besides, standardisation of protocols and phase III confirmatory trials are required to establish clinical benefit and support regulatory approval.

**Conclusion:**

tFGS holds considerable potential to transform surgical oncology in GI cancers by enabling more precise resections and guiding organ‐preserving strategies. Future innovations in multimodal tracers, NIR‐II fluorophores and theranostics may further enhance the precision and therapeutic potential of tFGS.

**Key points:**

- Tumour‐targeted fluorescence‐guided surgery (Ttfgs) enhances intraoperative precision in gastrointestinal cancer treatment.
- Translation of (Ttfgs) into clinical practice is limited by heterogeneity, regulatory hurdles, biomarker variability and absence of phase III trials.
- Innovations such as multimodal tracers and theranostic agents may further enhance the precision and therapeutic potential of Ttfgs.

## Graphical abstract

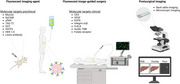

## 1 INTRODUCTION

Gastrointestinal (GI) cancers, including oesophageal, gastric and colorectal cancers (CRC), represent a significant global health burden, ranking among the leading causes of cancer‐related morbidity and mortality.^1^ Surgical resection remains the cornerstone of curative treatment for localised disease.^2^ However, achieving clear resection margins while preserving healthy tissue is challenging, particularly in cases of small, multifocal or poorly demarcated tumours. Traditional intraoperative assessment relies on visual inspection and palpation, which are limited in sensitivity, especially in minimally invasive surgery, such as laparoscopic or robot surgery. R0 resection rates vary from 40.1 to 98.9% for respectively pancreatic and gastric cancer and are a crucial indicator for survival.^3^, ^4^ Positive resection margins in CRC occur in an average of 12% of cases.^5^ Therefore, there is a clinical need for enhanced intraoperative visualisation techniques that support real‐time tumour localisation and resection guidance.^6^, ^7^

Fluorescence imaging has emerged as a promising solution to improve complete surgical removal of all tumour tissues. Fluorescence refers to the emission of light by fluorophores after excitation with a specific wavelength. After absorbing a photon, a fluorophore is promoted to an excited state. Following rapid non‐radiative relaxation, it returns to the ground state and emits a photon of lower energy, which can be detected with specialised imaging systems. Near‐infrared fluorescence (NIRF) imaging, particularly in the NIR‐I (700–900 nm) window, allows for deeper tissue penetration and minimal interference from tissue autofluorescence compared with the visible light spectrum, providing high‐contrast visualisation during surgery.^8^

One of the most widely used fluorophores in fluorescence‐guided surgery (FGS) is indocyanine green (ICG). Although it does not specifically target tumours, it is extensively used in GI surgery for assessing tissue perfusion, visualising lymphatics and aiding hepatobiliary procedures. Its safety and clinical utility have made ICG broadly applied in fluorescence imaging and surgical guidance.^9^

Building on this, tumour‐targeted fluorescence‐guided surgery (tFGS) is a rapidly evolving field enhancing intraoperative tumour localisation. In tFGS, fluorescent tracers are conjugated to tumour‐specific ligands, such as antibodies or peptides, and administered intravenously or topically prior to surgery. These tracers bind selectively to tumour cells or microenvironment, allowing for real‐time visualisation of malignant tissue under NIRF imaging.^10^ This approach supports multiple intraoperative applications: tumour localisation, identification of positive resection margins, detection of satellite lesions or multifocal disease, sentinel lymph node mapping and nodal staging, intraoperative detection of distant metastases and decision support in organ‐preserving strategies.

Despite promising preclinical and early‐phase clinical data, widespread clinical translation of tumour‐targeted tracers remains limited. Barriers include regulatory hurdles, standardisation of imaging platforms, variability in tumour biology, logistical challenges in tracer production and administration and means of reimbursement.

This systematic review aims to provide a comprehensive overview of tFGS evaluated in GI oncology surgery, discussing preclinical and clinical research. Furthermore, it explores current limitations and success factors for clinical implementation. And last, highlights future perspectives for advancing tFGS in routine surgical practice.

## 2 MATERIALS AND METHODS

The study protocol was registered with PROSPERO, ID number (558994) and Preferred Reporting Items for Systematic Reviews and Meta‐Analyses guidelines were followed.

### 2.1 Search strategy

A comprehensive search in databases PubMed and Embase was performed with a restriction on language. Only English studies were included. The last search was carried out on 17 September 2025. Search terms referring to GI tumours, tumour‐targeted biomarkers and fluorescent tracers were used in various configurations. Furthermore, the reference lists of selected articles were searched manually to identify additional studies. Details of our search strategy in PubMed and Embase are provided in the supplementary files.

### 2.2 Study selection

After removal of duplicates, two authors (A. D. and F. S.) independently screened the title and abstract of each record. The same authors independently assessed the full text of all eligible articles. Any disagreements between the authors were resolved via consensus or, in case of persistent discrepancies, by discussion with a third author (E. G.). Human and animal studies were included. Reviews, editorials and case reports were excluded. Molecular targets were included in the preclinical setting, if three or more articles discussed the molecular target.

### 2.3 Data extraction

Two reviewers (A. D. and E. G.) independently extracted data from the selected articles using a standardised form. Any disagreements were resolved by consensus. The following data were extracted: last name of the first author, full title, year of publication, study population, inclusion criteria, study size, molecular target, fluorophore, absorbance and emission wavelength, imaging system, dosage and timing of tracer administration, targeted organ and study outcomes, such as: tumour‐to‐background ratio (TBR)’s, sensitivity, specificity and alterations in clinical treatment plan.

### 2.4 Definitions

TBR is a quantitative measure in medical imaging that compares the intensity (fluorescence or radiotracer uptake) in a tumour to that in surrounding normal tissue (background). A higher TBR indicates better contrast between tumour and healthy tissue.

Sensitivity refers to the ability of a fluorescence‐guided imaging technique to correctly identify malignant tissue. It is defined as the proportion of true positive findings among all histopathologically confirmed malignant lesions. High sensitivity indicates a low rate of false‐negative results.

Specificity reflects the ability of an imaging modality to correctly identify non‐malignant tissue. It is defined as the proportion of true negative findings among all histopathologically confirmed benign or non‐tumourous tissues. High specificity indicates a low rate of false‐positive fluorescence signals.

Diagnostic accuracy refers to the overall ability of a fluorescence‐guided imaging technique to correctly classify tissue as malignant or non‐malignant. It is typically expressed as the proportion of correctly classified observations (true positives and true negatives) among all evaluated observations.

Alterations in the clinical treatment plan were defined as any intraoperative change in surgical decision‐making attributable to fluorescence imaging. This included, but was not limited to, extension or reduction of resection margins, additional lesion excision, modification of lymph node dissection or conversion to a different surgical strategy.

Back‐table imaging refers to ex vivo fluorescence imaging performed on the resected surgical specimen outside the patient, typically on a sterile back table. This technique is used to assess margin status, tumour localisation or specimen orientation and may guide additional intraoperative surgical actions when residual tumour involvement is suspected.

## 3 RESULTS

**FIGURE 1:**
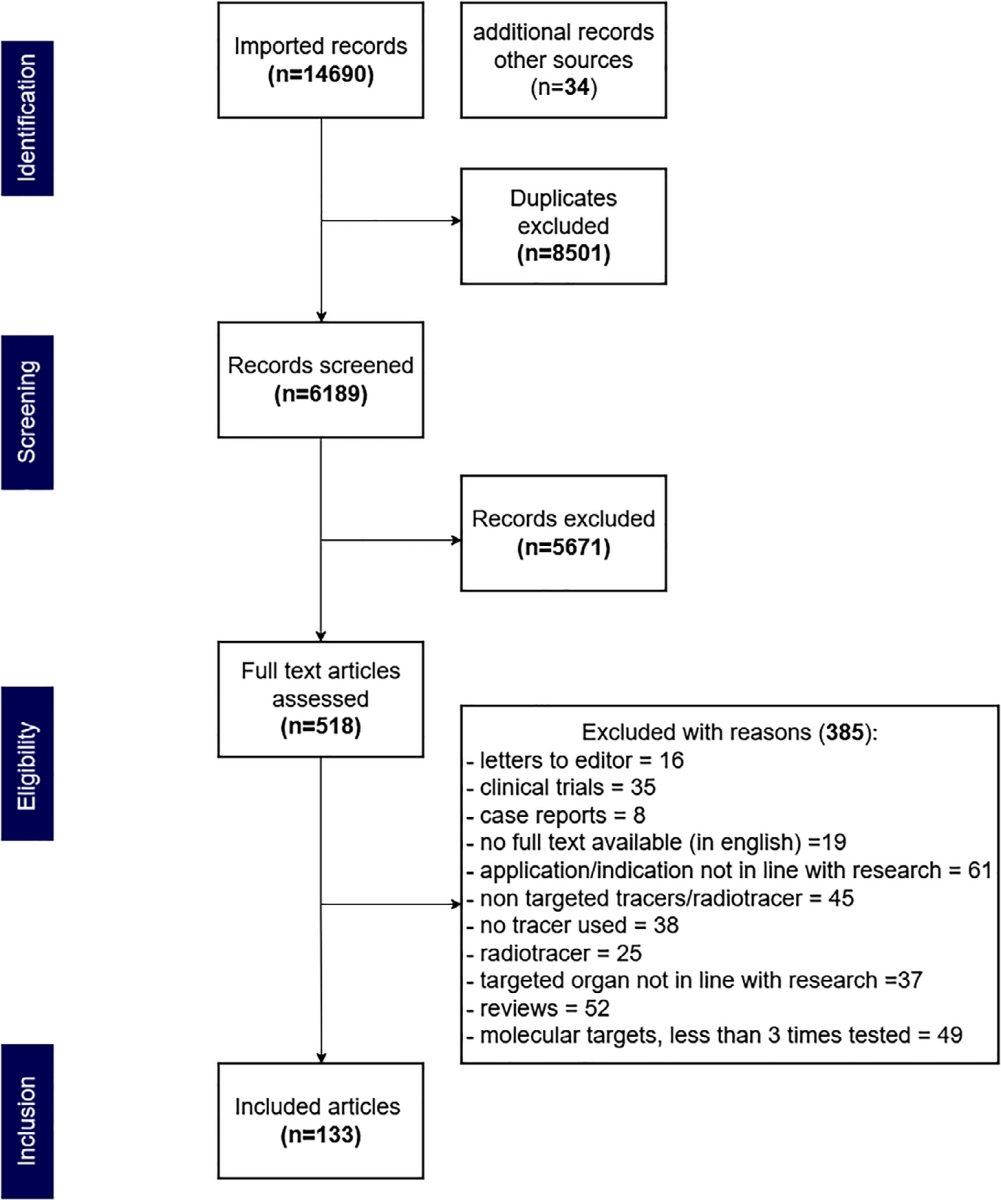
Flow chart diagram included articles.

**FIGURE 2:**
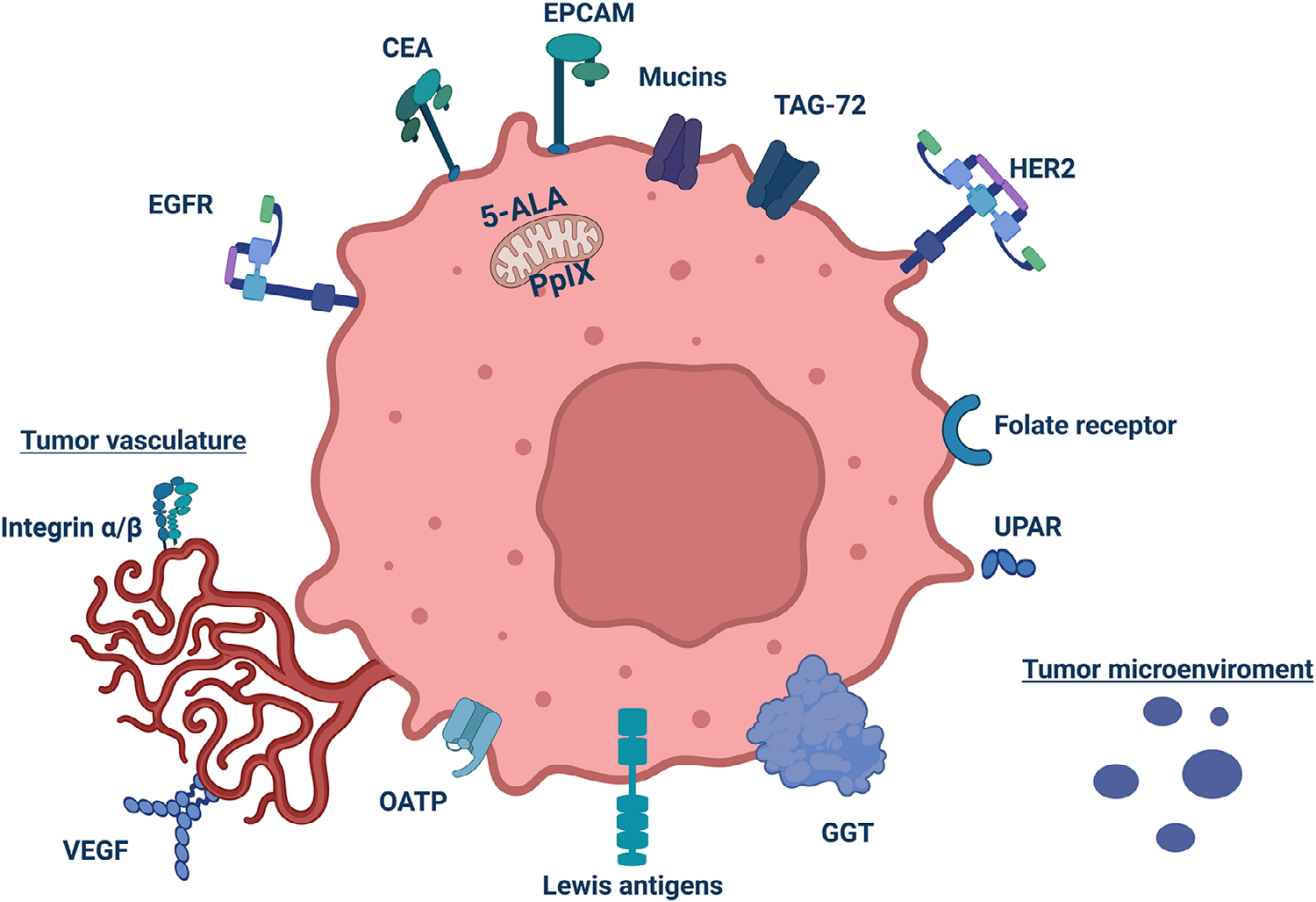
Localisation of molecular targets on the cancer cell membrane, intracellularly, and within the tumour microenvironment. *Abbreviations*: CEA, carcinoembryonic antigen; UPAR, urokinase receptor; TAG‐72, tumour‐associated glycoprotein 72; HER2, human epidermal growth factor receptor 2; EPCAM, epithelial cell adhesion molecule; GGT, gamma‐glutamyltranspeptidase; OATP, organic anion transporting polypeptide; EGFR, epidermal growth factor receptor; VEGF, vascular endothelial growth factor. Figure 2 was created with Biorender.com.

**FIGURE 3:**
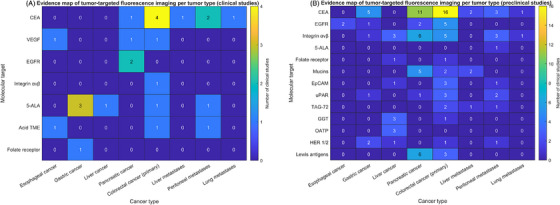
Evidence map of tumour‐targeted fluorescence imaging per molecular target and tumour type in (A) clinical studies and (B) preclinical studies. *Abbreviations*: CEA, carcinoembryonic antigen; UPAR, urokinase receptor; TAG‐72, tumour‐associated glycoprotein 72; HER2, human epidermal growth factor receptor 2; EPCAM, epithelial cell adhesion molecule; GGT, gamma‐glutamyltranspeptidase; OATP, organic anion transporting polypeptide; EGFR, epidermal growth factor receptor; VEGF, vascular endothelial growth factor. Figure 3 was created with MATLAB version: 23.2.0. (R2023b), Natick, Massachusetts: The MathWorks Inc.; 2023.

A total of 133 articles were included in this review as shown in Figure 1. Tracers tested in clinical trials are discussed first, followed by evaluation of preclinically investigated targets. Figure 2 shows a graphical overview of all targets and their (extra)cellular localisation. Figure 3 visualises an evidence map of the molecular targets and tumour types in pre‐ and clinical trials. Last, challenges regarding clinical translation are addressed, together with future perspectives on tracer development, clinical validation and implementation.

### 3.1 Tumour‐targeted fluorescent tracers evaluated in humans

In total, nine tracers have been tested in clinical trials in GI oncology surgery, targeting different biomarkers in different GI tumours or the tumour microenvironment (TME). These targets are discussed in detail below; an overview of the study results can be found in Table 1.

**TABLE 1:** In human tested tFGS tracers, study size and outcomes.

Fluorescent agent molecular target fluorophore	Excision–emission wavelength	Clinical imaging system	Study size—target organ(s)	Administration—dosage and timing	Study outcome
SGM‐101 CEA BM‐104	685–705 nm	Quest Spectrum® (Quest Medical Imaging, Middenmeer, the Netherlands) Quest Artemis (Quest Medical Imaging, Middenmeer, the Netherlands)	*N* = 12—PDAC	5, 7.5, 10 mg 48–96 h before surgery	The mean TBR primary tumour = 1.6, metastatic lesions 1.7; one false‐positive lesion was detected (CEA‐expressing intraductal papillary mucinous neoplasm). Two false‐negatives: overlying blood or tissue that blocked the fluorescent signal.^6^
		Quest Spectrum® (Quest Medical Imaging, Middenmeer, the Netherlands) Quest Artemis (Quest Medical Imaging, Middenmeer, the Netherlands)	*N* = 26—CRC	5, 7.5, 10 mg 48–96 h before surgery	Mean TBR 6.1, 35% changed treatment strategy. Sensitivity 98%, specificity 62%^12^
		Quest Spectrum® (Quest Medical Imaging, Middenmeer, the Netherlands)	*N* = 37—CRC	5, 7.5, 10, 12.5, 15 mg 24–72 h before surgery	2 false‐positive. Mean TBR 1.9 in 10 mg group. sensitivity 96% spec. 63% NPV 94%. 24% surgical plan alteration^51^
		Quest Spectrum® (Quest Medical Imaging, Middenmeer, the Netherlands)	*N* = 12—CRC	96–144 h before surgery	103 lesions sensitivity 98.5% specificity 62.2%; 14 lesions false‐positive PPV 82.3% NPV 95.8%^53^
		Quest Spectrum® (Quest Medical Imaging, Middenmeer, the Netherlands)	*N* = 11—liver CRC	5–15 mg 48–96 h before surgery	17 of 19 suspected lesions were malignant, mean TBR 1.7. 2 false‐positive^52^
		Quest Spectrum® (Quest Medical Imaging, Middenmeer, the Netherlands)	*N* = 13—CLM CRC	7.5, 10, 12.5 mg 72–120 h before surgery	TBR in vivo 1.00, back table 1.45, closed‐field bread loaf imaging 4.81 positive fluorescence signal resp: 31, 45, 94%^54^
[^111^In]In‐DOTA–labetuzumab–IRDye800CW/111In‐DOTA–hMN‐14–IRDye800CW CEA IRDye–800CW	778–794 nm	Quest Spectrum® (Quest Medical Imaging, Middenmeer, the Netherlands)	*N* = 15—PM CRC	5, 10, 50 mg 120–148 h before surgery	Dose escalation 2 (*n* = 5), 10 (*n* = 5), 50 (*n* = 5) mg. 10 mg for ideal doses. Mean SPECT/CT PCI was 3 (SD 3), mean clinical PCI of 10 (SD 6) (*p* < .001). alteration of clinical strategy in 30% (*n* = 3)^55^
		Odyssey® (LI‐COR Biosciences, Lincoln, NE, USA)	*N* = 10—CRC	20 mg	Fluorescence intensity was higher in tumour areas compared with non‐tumour tissue parts (*p* < .001). Average fluorescence tumour‐to‐background ratio was 11.8 ± 9.1:1^56^
		Quest Spectrum® (Quest Medical Imaging, Middenmeer, the Netherlands)	*N* = 7—PM CRC	10 mg 120–148 h before surgery	With NIRF 34 (92%) of 37 malignant lesions were detectable. Of 52 fluorescent lesions, 4 were false‐positive. Mean TBR was 3.4 (1.8) and mean radiodetection TBR was 4.4(1.4).^57^
Bevacizumab–irdye800cw VEGF‐A IRDye–800CW	778–795 nm	EXPLORER AIR® (SurgVision GmbH, Munich, Germany)	*N* = 10—susp. PDAC	4.5, 10, 25 mg 72 h before surgery	Dose escalation phase I 4.5 (*n* = 4), 10 (*n* = 3) or 25 mg (*n* = 3). Ex vivo TBRs were 1.3, 1.5 and 2.5 for the 4.5‐, 10‐ and 25‐mg groups, resp.^58^
		EXPLORER AIR® (SurgVision GmbH, Munich, Germany)	*N* = 20—oesophageal cancer	4.5, 10, 25 mg 48–72 h before surgery	4.5 (*n* = 5), 10 (*n* = 5) or 25‐mg bevacizumab–800CW. TBR 1.33, 2.13 and 2.98 in 4.5‐, 10‐ and 25‐mg^60^
		EXPLORER AIR® (SurgVision GmbH, Munich, Germany)	*N* = 25—LARC	4.5 mg 48–72 h before surgery	4.5 mg of bevacizumab–800CW. FGI identified a tumour‐positive CRM by high fluorescence intensities in 1 of 2 patients (50%) 5 of 6 tumour‐negative CRM (83%). Microscopic TBR 4.7^59^
Cetuximab–IRDye800 EGFR IRDye–800CW		EXPLORER AIR® (SurgVision GmbH, Munich, Germany)	*N* = 7—PDAC	50, 100 mg	Significantly higher mean fluorescence intensity in the tumour (.09 ± .06) vs. normal pancreatic tissue (.02 ± .01), vs. pancreatitis (.04 ± .01; *p* < .001) sens. 96.1% and spec. 67.0%^71^
Panitumumab–IRDye800CW EGFR IRDye–800CW		SPY‐PHI® (Stryker, Michigan, USA) EXPLORER AIR® (SurgVision GmbH, Munich, Germany)	*N* = 11—PDAC	Loading dose of 100 mg panitumumab + additional 25, 50, 100 mg of fluorescent labelled panitumumab 48–120 h before surgery	TBR = 3, 4 and 3.7 for respectively 25, 50 and 75 mg group (primary PDAC tumour) Sensitivity = 90% Specificity = 74%^72^
cRGD–ZW800‐1 Integrin αvβ3 ZW800‐1	785–805–850 nm	Visera Elite (Olympus, Tokyo, Japan) Quest Spectrum® (Quest Medical Imaging, Middenmeer, the Netherlands)	*N* = 11—healthy *N* = 12—colon cancer	.005, .015, .05 mg/kg 2–18 h before surgery	Non‐related AEs/SAEs. Ideal dose .05 mg/kg 18 h TBR = 1.1–16 (2–4 and 18 h)^91^
5‐Aminolevulinic acid Cellular uptake in tumour cells protoporphyrin IX (PpIX)	400–662 nm	Xenon short‐arc lamp (D‐Light, Karl Storz, Germany)	*N* = 6—gastric cancer	20 mg/kg of 5‐ALA 6 h before surgery/endoscopy	Sensitivity = 81% peritoneal carcinomas Sensitivity = 60% gastric cancer^93^
		Endoscopic PDDsystem (Karl Storz, Tuttlingen, Germany) including a CCU TricamSLII/3CCD CH Tricam‐P PDD, HOPKINS II Straight Forward Telescope 30° (Karl Storz)	*N* = 21—gastric cancer	1.0 g 3–4 h before surgery	Sensitivity = 57.7% Specificity = 100% Diagnostic accuracy = 66.7%^94^
		Sie‐P video image endoscope (Fuji Film Medical Co., Tokyo, Japan)	*N* = 41—gastric cancer	20 mg/kg 3 h before endoscopic resection	Sensitivity 85.3% Specificity 91.7%; Detection rate of 86.5%^95^
		Esperaluz (CCS Inc. Kyoto, Japan) Sie‐P video image endoscope (Fuji Film Medical Co., Tokyo, Japan)	*N* = 70—HCC	1.0 g 3 h before surgery	100% detection of HCC, 85.7% detection of CRCLM^96^
		Visera Elite (Olympus, Tokyo, Japan)	*N* = 12—CRC	15–20 mg/kg 3 h before surgery	Better diagnostic accuracy using 5‐ALA‐PDD compared with conventional laparoscopy in patients with colorectal cancer^97^
		Xenon lamp (300 W) with blue light with a 375–445 nm	*N* = 115—PM	20 mg/kg 4 h before surgery	Overall detection rate 35.6%; 54% crc, 17% appendiceal mucinous neoplasms, 33% gastric cancers^98^
ONM‐100 Acidic TME NIR dye in polymer micelles	750–830 nm	EXPLORER AIR® (SurgVision GmbH, Munich, Germany) SPY‐PHI® (Stryker, Michigan, USA)	*N* = 3—oesophageal cancer *N* = 3—CRC	.3, .5, .8, .1, 1.2 mg/kg 24 ± 8 h before surgery	1.2 mg/kg, TBR = 4.5^99^
		EleVision™ IR Platform (Medtronic, Minneapolis, USA) EXPLORER AIR® (SurgVision GmbH, Munich, Germany) Pdeneo (Hamamatsu Photonics K.K., Shizuoka, Japan)	*N* = 40—peritoneal carcinomatosis (6 different primary tumours)	1 mg/kg 24–72 h before surgery	Residual disease was detected in 20 of 40 (50%) patients 32.5% had a change in cytoreductive score based on NIRF imaging. Sensitivity = 78.6% Specificity = 56.4%^100^
OTL38 folate receptor alpha (FRa) S0456	774–776 to 794–796 nm	EleVision™ IR Platform (Medtronic, Minneapolis, USA)	*N* = 5—gastric cancer	.025 mg/kg 1.5–6 h before surgery	3/4 patients gastric resection fluorescent mean TBR of 4.1^103^

Targeting ligands include monoclonal antibodies and smaller molecules such as peptides, nanobodies, antibody fragments, designed ankyrin repeat proteins (DARPins) and nanoparticles (NPs). Depending on the target ligand, the half‐life of the tracer differs, which influences the timing of administration. Monoclonal antibodies show high specificity, however, entail logistical challenges. Due to their large size several limitations arise, including reduced tumour penetration, delayed imaging windows and excessive costs. Smaller molecules are suggested to overcome these limitations. Consequently, the included clinical studies represent a heterogeneous spectrum of targeting ligands with distinct pharmacokinetic and imaging properties, which is considered when comparing administration strategies and reported outcomes.^11^

#### 3.1.1 Carcinoembryonic antigen

Carcinoembryonic antigen (CEA) is known as a tumour target for CRC and is expressed in 90% of primary colorectal tumours.^12^ Overexpression of CEA is also seen in pancreatic, oesophageal cancer, peritoneal, colorectal liver and lung metastases. CEA has been extensively tested as tumour target in animals with CEA‐antibodies linked to different fluorophores, such as IRDye–800CW,^13^, ^14^, ^15^, ^16^, ^17^, ^18^, ^19^, ^20^, ^21^, ^22^, ^23^, ^24^, ^25^, ^26^, ^27^, ^28^, ^29^, ^30^, ^31^, ^32^ Alexa 488/555/647,^33^, ^34^, ^35^, ^36^, ^37^, ^38^, ^39^, ^40^, ^41^ DyeLight750/650,^42^, ^43^ BM‐104^44^, ^45^, ^46^, ^47^ and others,^48^, ^49^, ^50^ as shown in Table S1.

SGM‐101 is a chimeric monoclonal antibody (mAb) targeting CEA conjugated to the NIR fluorophore BM‐104. After extensive preclinical testing, SGM‐101 was first tested in humans in a dose escalating study in nine CRC patients and an expansion cohort of 17 patients that were administered the optimal dose of 10 mg.^12^ A diagnostic accuracy of 84% was found. Another expansion of the study evaluated the use of SGM‐101 in a larger cohort (*n* = 37).^51^ All patients received SGM‐101, and fluorescence signals were visible in all primary and recurrent tumours. In 24% of the case, the clinical strategy was altered. Furthermore, the safety and feasibility of SGM‐101 for use in primary and metastatic pancreatic cancer (PDAC) was investigated in a dose escalation phase I study in 12 patients. One false‐positive lesion was detected, and a TBR of 1.6 (in vivo) and 3.2 (ex vivo) of the primary tumours was reported. The mean TBR in metastatic lesions was 1.7.^6^ SGM‐101 has also successfully been investigated for the detection of colorectal and pancreatic liver metastases.^52^ SGM‐101 was also tested in patients undergoing cytoreductive surgery with hyperthermic intraperitoneal chemotherapy for metastatic CRC. The main objective was to distinguish whether the peritoneal carcinomatosis index (PCI), and thus the completeness of cytoreduction, can be changed with SGM‐101 and fluorescence imaging. A correct change in PCI owing to fluorescence imaging was seen in four of the 14 included patients.^53^ Last, SGM‐101 has also been successfully investigated in lung metastases.^52^, ^54^

[^111^In]In‐DOTA–labetuzumab–IRDye800CW and ^111^In‐DOTA–hMN‐14–IRDye800CW combine a radionuclide ([^111^In]Indium) and a NIRF dye (IRDye800CW) conjugated to an anti‐CEA antibody (labetuzumab) to enhance depth penetration. Pre‐clinical studies have demonstrated the safety and potential in improving tumour localisation during surgery.^20^, ^21^, ^32^ A phase I clinical trial exhibited the potential of multimodal imaging to detect PM of colorectal origin. In the optimal dose (10 mg) group, 95% of all malignant lesions could be detected and 16% were false‐positive caused by either inflammation or fibrosis. In 30% of cases, the clinical strategy was altered and previously undetected lymph nodes were detected.^55^, ^56^ These outcomes were confirmed in a confirmation trial in patients with PM of colorectal origin.^57^

#### 3.1.2 Vascular endothelial growth factor

Bevacizumab–IRDye800CW consists of an anti‐vascular endothelial growth factor (VEGF) antibody conjugated with IRDye800CW. Elaborate preclinical research has been conducted, however, not in GI oncology. A multicentre dose escalating study of bevacizumab–IRDye800CW was performed in 10 patients who were clinically suspected of PDAC. Results demonstrated suboptimal TBRs as no distinguishment was possible between tumourous and inflamed tissue due to the desmoplastic stroma, poor vascularisation and high intertumoural pressure tracer accumulation is hampered. Therefore, bevacizumab–IRDye800CW is not a suitable tracer in PDAC patients.^58^ In locally advanced CRC, however, bevacizumab–IRDye800CW did demonstrate potential for clinical implementation: NIR back‐table imaging of CRC surgical specimens for margin assessment and presence of colorectal metastases (CRM) was evaluated.^59^ Back‐table NIRF imaging correctly identified five out of six tumour negative CRM potentially preventing unnecessary additional rection in one patient. Furthermore, the use of bevacizumab–IRDye800CW was investigated for NIR endoscopic neoadjuvant treatment response evaluation prior to surgery in 20 oesophageal cancer patients. Bevacizumab–IRDye800CW was able to differentiate between cancerous tissue and healthy oesophageal tissue.^60^ However, differentiation between a complete response and presence of residual tumour cells was not possible.

#### 3.1.3 Epidermal growth factor receptor

Cetuximab–IRDye800CW is a chimeric anti‐epidermal growth factor receptor (EGFR) antibody, conjugated with IRDye800 for NIRF imaging. Cetuximab and other EGFR inhibitors were preclinically tested in mouse models as discussed in Table S3.^49^, ^61^, ^62^, ^63^, ^64^, ^65^, ^66^, ^67^, ^68^, ^69^, ^70^ Clinical trials have explored its use in various cancers, but only two in GI cancers.^71^, ^72^ A dose‐escalation study of cetuximab–IRDye800 was performed in seven PDAC patients to evaluate the safety and feasibility of intraoperative tumour identification. NIRF imaging after cetuximab–IRDye800 administration could differentiate between pancreatitis and PDAC. With a mean fluorescence intensity in the tumour (.09 ± .06) versus normal pancreatic tissue (.02 ± .01; *p* < .001) was found. A sensitivity of 96.1% and a specificity of 67.0% was found.^71^ Due to infusion reactions of cetuximab, the same group developed another anti‐EGFR antibody; panitumumab. Panitumumab–IRDY800CW has an improved safety profile and was tested in 11 PDAC patients. The optimal dose of 50 mg resulted in a TBR of 4.^72^

#### 3.1.4 Integrin αvβ

cRGD–ZW800‐1 targets predominantly integrin avβ3, overexpressed in tumour angiogenesis. cRGD–ZW800‐1 has undergone trials to assess its safety and efficacy in tumour imaging in murine models as discussed in Table S4.^73^, ^74^, ^75^, ^76^, ^77^, ^78^, ^79^, ^80^, ^81^, ^82^, ^83^, ^84^, ^85^, ^86^, ^87^, ^88^, ^89^, ^90^ The results indicated potential for intraoperative visualisation of tumours with high angiogenic activity, including GI malignancies. De Valk et al. performed the first in‐human clinical trial of cRGD–ZW800‐1 in 12 CRC patients and eleven control subjects.^91^ In vivo TBRs ranging from 1.1 to 1.6 were found after tracer administration of 2–4 h, respectively. Significant differences were reported between ex vivo TBRs of tumour and mucosa.

#### 3.1.5 5‐Aminolvulinic acid

5‐Aminolvulinic acid (5‐ALA) is a prodrug metabolised into protoporphyrin IX, a fluorescent compound accumulating in tumour cells, by blue light irradiation (380–450 nm). While primarily used for glioblastoma resection, a preclinical study has investigated its application in GI cancers as discussed in Table S5.^92^

5‐ALA was first clinically used in GI oncology to detect early gastric cancer in six patients yielding a sensitivity of 60%.^93^ False‐positives were most likely caused by inflammation. Detection of PM in diagnostic laparoscopy resulted in a sensitivity of 81%. Fluorescence signals were two to four times higher in malignant tissues compared with normal mucosa. In addition, a study concerning a larger gastric cancer cohort of 21 patients was performed. An accuracy of 66.7% and a sensitivity of 57.7% was found.^94^ Recently, a clinical study was performed in 43 intestinal types of gastric cancer patients.^95^ Forty‐five out of 52 lesions were detected in vivo by 5‐ALA yielding a detection rate of 86.5%. Furthermore, 5‐ALA was used in 70 liver resections.^96^ A detection rate of 100% in primary HCC and 85.7% in CRC liver metastases was found. 5‐ALA is also feasible to detect PM of CRC origin. One out of 12 patients had a lesion detected by 5‐ALA which was undetected by white light imaging.^97^ A larger cohort study, including 115 patients, revealed an overall detection rate of 35.6%. PM of CRC origin yields a detection rate of 54%.^98^

#### 3.1.6 Acidic TME

ONM‐100 is a pH‐sensitive micelle‐based NP conjugated with a NIR dye, designed to target the acidic TME) of tumours. Elaborate preclinical research has been performed, however, not in GI oncology. A phase I study was performed in 30 patients with a variety of solid tumours to evaluate the safety, pharmacokinetics and imaging feasibility of ONM‐100 conjugated to ICG. A total of three oesophageal and three CRC patients were included. Four additional lesions were detected which would otherwise be unnoticed using white light imaging.^99^ A drawback of ONM‐100 is the activation by other mechanisms associated with a lowered pH, for example, inflammation. Furthermore, a phase II trial was conducted to evaluate ONM100 for detection of residual PM during cytoreductive surgery.^100^ In total, 40 patients were included, and only areas of residual disease identified with NIRF imaging were resected after routine cytoreductive surgery. A total of 20 patients presented with residual disease of which 32.5% had a change in cytoreductive score based on NIRF imaging. Sensitivity and specificity were 78.6 and 56.4%, respectively.

#### 3.1.7 Folate receptors

Pafolacianine, sold under the brand name Cytalux (OTL‐38), targets folate receptor (FR) α and β which are responsible for transport of folate into cells. High expression of FRβ has been demonstrated in tumour‐associated macrophages. Preclinical studies demonstrate potential for clinical translation as discussed in Table S6.^101^, ^102^ A clinical trial was initiated by Newton et al. to investigate OTL38 in five gastric adenocarcinoma patients. OTL38 is composed of NIR dye S0456 conjugated to a folate analogue. Three out of the four patients subjected to a (sub)total gastrectomy showed a fluorescent positive tumour with a mean TBR of 4.1. In one patient the tumour foci were relatively too small to detect (<.4 cm).^103^ Pafolacianine has received United States Food and Drug Administration (US FDA) approval for its use in cancer surgery, specifically targeting ovarian and lung cancer. This approval highlights its effectiveness in intraoperative detection of malignant tissues, aiding in the complete resection of tumours during surgical procedures.^104^, ^105^

### 3.2 Preclinical evaluation of tumour‐targeted fluorescent tracers

A wide variety of molecular targets and tracers were tested in the preclinical setting in animal models and are pending clinical translation. Another 39 targets were identified. Discussed are the eight high potential targets as described in *Materials and Methods* section. An overview of these preclinical studies is found in Tables S7–S13 based on molecular targets.

#### 3.2.1 Mucins

Mucins are glycoproteins normally expressed in glycosylated form in the stomach, gallbladder and GI tract lumen, but absent in the pancreas, liver and GI serosa. Non‐glycosylated mucins are overexpressed in various cancers. A MUC1‐targeting NIR probe was developed for intraluminal detection of small CRC lesions, showing threefold higher intensity compared with non‐targeted copolymers in CRC tissues and mouse models.^106^ Similarly, a MUC4 antibody was used to target CRC and liver metastases, yielding TBRs of 2.17 and 2.56 in primary and metastatic patient‐derived orthotopic xenograft (PDOX) models, respectively.^107^ This was also tested targeting human pancreatic cancer in orthotopic cell line mouse models; high tumour‐to‐liver ratios of 2.74 (±.174) and 2.73 (±.223) in the SW1990 and CD18/HPAF orthotopic models, respectively, were obtained.^108^ Last, a mucin‐4 targeting tracer achieved a TBR of 2.42 and TLRs as high as 9.05 in pancreatic cancer liver metastasis mouse models.^109^ In PDAC, mucin‐5AC, mucin‐16 and mucin‐4 were identified as viable targets. Mucin‐16 is overexpressed in 60–80% of PDAC. The tracer huAR9.6–IRDye800 demonstrated high specificity, and a TBRs > 3, with a peak of 6.95.^110^, ^111^ MUC5AC–IR800 showed potential in PDAC and liver metastases, achieving TBRs of 4.35 and up to 7.034, respectively.^112^, ^113^ While these findings support mucins as promising targets for tFGS, further research in diverse cancer populations is needed to support clinical translation. Table S7 shows an overview of preclinical studies.

#### 3.2.2 Epithelial cell adhesion molecule

Epithelial cell adhesion molecule (EpCAM) is involved in cellular signalling and plays a role in tumour cell migration, proliferation and differentiation, making it a promising target for tFGS. An EpCAM mAb conjugated to IRDye800CW achieved a TBR of 13.5 in a CRC PDOX model.^114^ However, mAbs have limitations due to their large size, including reduced tumour penetration, delayed imaging windows and higher costs. To overcome these, smaller alternatives such as anti‐EpCAM Fabs and DARPins have been evaluated.^115^, ^116^ Though these have lower stability and affinity than mAbs, they still yielded strong imaging results with TBRs of 11.5 (Fab) and 4.2 (DARPin). Additionally, EpCAM‐targeting NPs have been explored as triple‐modality agents for both diagnosis and therapy in liver cancer.^117^ These NPs may aid in non‐invasive management of small liver tumours. Given EpCAM's relevance across various cancer types, it represents a highly promising tumour‐specific target. Table S8 shows an overview of preclinical studies.

#### 3.2.3 Urokinase‐type plasminogen activator receptor

Urokinase‐type plasminogen activator receptor (uPAR) is up‐regulated in most carcinomas, including CRC and PDAC cancers, and is associated with tumour invasion, angiogenesis and metastasis. Boonstra et al. proofed the feasibility of an uPAR‐specific mAb (ATN‐658) multimodal targeted agent conjugated to ZW800‐1 and radionuclide 111‐indium, reporting TBRs of 5.0 in CRC PDOX models and 1.3 in controls, supporting effective tumour delineation and preoperative SPECT imaging.^118^ In PDAC, Juhl et al. evaluated intraoperative NIRF imaging using an uPAR‐targeted probe (ICG–Glu–Glu–AE105), achieving TBRs of 3.5 in primary tumours and 3.4 in metastases^119^ NIRF imaging identified 14% more metastases than white light surgery, with additional lesions detected in four of eight mice. To address the limitations of mAbs, Fabs were tested as alternative probes in CRC PDOX models. Although imaging could be performed earlier, peak fluorescence intensity was lower; TBRs remained above two.^120^ In addition, Mateusiak et al. demonstrated significantly higher TBRs in a variety of cancers using a nanobody‐based anti‐uPar tracer compared with the control group.^121^ Table S9 shows an overview of preclinical studies.

#### 3.2.4 Tumour‐associated glycoprotein‐72

Tumour‐associated glycoprotein (TAG)‐72 is a pancarcinoma antigen overexpressed in approximately 80% of CRC. In 2018, Gong et al. evaluated a single‐chain variable fragment of 3E8 mAb conjugated to IRDye800 in an orthotopic CRC model.^122^ The tracer demonstrated favourable pharmacokinetics, safe biodistribution and strong binding specificity in six mice. Hollandsworth et al. conducted two preclinical studies using a humanised anti‐TAG‐72 antibody (anti‐huCC49) conjugated to IRDye800 for detecting CRC liver metastases. The first study optimised synthesis, dosing and timing, achieving a maximum TBR of 7.39 at 72 h post‐injection of 50 µg tracer. This enabled visualisation of both regional and PM that were otherwise undetectable.^123^ The second study, therefore, served as a proof‐of‐concept that the tracer can image CRC liver metastases. The second study confirmed these findings in metastatic models, reporting a mean intra‐vital tumour‐to‐liver ratio of 7.43.^124^ Table S10 shows an overview of preclinical studies.

#### 3.2.5 Gamma‐glutamyltranspeptidase

Gamma‐glutamyltranspeptidase (GGT) is a membrane‐bound enzyme involved in the metabolism of glutathione, playing a crucial role in cellular detoxification and protection against oxidative stress. Elevated levels of GGT are often associated with various cancers, making it a valuable biomarker for diagnosis and prognosis. Miyata et al. evaluated a topical GGT‐targeting probe on 103 freshly resected human hepatic cancer specimens, reporting sensitivities of 48% (HCC), 87% (CRC liver metastases) and 100% (intrahepatic cholangiocarcinoma), with specificities of 96–100%.^125^ GGT expression may also predict postoperative recurrence, as higher levels are linked to microscopic vascular invasion. Lui et al. Developed a ‘AIE + ESIPT’ near‐infrared nanoprobe designed for imaging GGT in cells, utilising aggregation‐induced emission and excited‐state intramolecular proton transfer mechanisms to enhance fluorescence upon interaction with GGT. It showed high specificity and stability, making it suitable for clinical applications. The probe exhibited a linear response to GGT concentrations and was successfully tested in HepG2 cells.^126^ Zhang et al. introduced poly‐g‐BAT, a tracer usable by injection or spray, tested in CRC surgical specimens and PDOX models. The probe showed a TBR of up to 12.3 after topical administration and enabled detection of premalignant lesions under 1 mm.^127^ Yang et al. also developed a GGT‐targeting tracer which can be administered both systemically and topically. A 9.8‐fold higher fluorescence intensity for tumour‐bearing mice was found compared with the control group.^128^ Table S11 shows an overview of preclinical studies.

#### 3.2.6 Organic anion‐transporting peptides

Organic anion‐transporting peptides (OATPs) are membrane‐bound transport proteins that facilitate the uptake of various endogenous and exogenous compounds, including drugs and imaging agents, into cells. They are particularly significant in cancer imaging due to their overexpression in many tumour types, which enhances the intracellular accumulation of imaging agents. OATP is researched in HCC to function as an alternative to molecular agents. An et al. demonstrate the effectiveness of an OATP probe in HCC imaging.^129^ A sixfold increase in fluorescence signal was found in HCC compared with liver cirrhosis. In addition, a significant correlation was found between NIR dye uptake and tumour cells. Song et al. show evidence that OATP indeed mediates tumour‐specific accumulation and retention of the tracer in HCC.^130^ Compared with ICG, a twofold higher TBR was found. Last, Rietbergen et al. conjugated a radionuclide to an OATP targeting tracer to enable multimodal imaging in a porcine model.^131^ Table S12 shows an overview of the preclinical studies.

#### 3.2.7 Human Epidermal Growth Factor Receptor 1/2

Overexpression of human EGFR (HER1) and HER2 drives tumour progression, and fluorescent tracers targeting these receptors enable real‐time surgical navigation. In the lapatinib‐based small molecule series YQ H(01–07), the probe YQ H06 showed a high tumour‐to‐normal ratio in orthotopic colorectal and hepatic models, providing strong contrast.^132^ A two‐step HER1 imaging strategy involves pre‐targeting tumours with biotinylated cetuximab followed by the application of neutravidin BODIPYFL, which binds to the antibody and amplifies the signal 10‐fold, allowing detection of PM ≥ .8 mm with 96 sensitivity and 98% specificity.^133^ For HER2, trastuzumab IRDye800CW exhibits increasing tumour signals up to 72 h. Dual‐labelling trastuzumab with IR700 and IR800 enables pre‐therapy imaging with a TBR of 6.2–6.7, facilitating precise localisation of HER2‐positive tumours before surgical intervention.^134^ Dual‐labelling trastuzumab‐IR700 with a second dye (IRDye800CW) enabled real‐time visualisation of HER2‐positive tumours without compromising NIR‐PIT efficacy. In mice, high contrast was delivered (TBR ≈ 161) versus small‐animal imaging (TBR ≈ 6–7), fluorescence fell after PIT as expected.^135^ These probes advance FGS, enhancing the ability to achieve complete tumour resection and improve surgical outcomes in cancers overexpressing HER1 and HER2.^131^ Table S13 shows an overview of the preclinical studies.

#### 3.2.8 Lewis antibody

CA19‐9, known as sialyl Lewis^a^, is a ligand for epithelial–leukocyte adhesion molecules, and it is associated with several different types of GI cancers. Anti‐CA19‐9 conjugated to AlexaFluor488 demonstrated in vivo tumour binding in a pancreatic cancer orthotopic mouse model and revealed microscopic foci not visible by white light imaging.^136^ Anti‐CA19‐9 conjugated to DyeLight 650 was used to determine the efficacy of neoadjuvant chemotherapy in combination with FGS. The metastatic recurrence frequency was reduced compared with solely FGS and white light imaging in a metastatic pancreatic cancer mouse model.^137^ Anti‐CA19‐9 coupled to IRDye800CW, tested in PDAC orthotopic mouse models, demonstrated significantly higher tumour‐to‐pancreas and ‐liver ratios compared with the control group using the PEARL and a clinical NIR imaging system.^138^ In addition, a dual‐labelled PET/NIRF anti‐CA19.9 tracer was developed which showed adequate metastases delineation and mapping of sentinel lymph nodes.^139^ To overcome the challenges associated with long‐lived radionuclides in dual‐labelled tracers, Adumeau et al. developed a method to decouple the radionuclide and anti‐CA19‐9 antibody at the time of injection.^140^ The use of a dual‐labelled short‐lived radionuclide tracer provided both PET imaging and NIRF‐guided excision of CRC tumours. The anti‐Lewis^a/c/x^ glycan antibody CH88.2, labelled with IRDye800CW, enabled in vivo tumour visualisation. In mice bearing CRC and pancreatic tumours, tumours were clearly delineated, with optimal imaging around 96 h (TBR ∼3.1 on Pearl; ∼2.2 on the clinical Artemis system for CRC; ∼1.8 for pancreatic cancer).^141^ Similarly, CH88.2–800CW and CH129–800CW were evaluated for bimodal NIRF/PA imaging^142^ A TBR of 4.8 ± 1.4 (CH88.2–800CW) and 4.9 ± .5 (CH129–800CW) in a CRC mouse model and a TBR of 2.5 ± .3 (CH88.2–800CW) and 2.9 ± .4 (CH129–800CW) in a pancreatic cancer were found. These findings demonstrate the feasibility of glycan‐targeted NIRF tracers for FGS. Table S14 shows an overview of the preclinical studies.

### 3.3 Challenges in clinical translation

Research on tumour‐targeted tracers is hampered by substantial financial and logistical demands, as well as biological challenges such as tumour heterogeneity. Moreover, comparison across studies remains difficult due to variability in imaging equipment, tracer dosage and timing of administration, which undermines reproducibility and complicates interpretation of results. The absence of standardised evaluation methods further limits cross‐study comparability, ultimately slowing the development of broadly applicable clinical guidelines and the establishment of reimbursement frameworks.^143^

Testing the toxicity and stability of tracers is both costly and time consuming. Clinical translation is further complicated by nonlinear tracer biodistribution, which can arise from receptor saturation or variations in tumour biology, making it challenging to extrapolate findings across different doses or patient populations. In this context, tracers with faster pharmacokinetics, such as Fabs, nanobodies and peptides, are advantageous.^11^ These tracers can be administered closer to surgery, offering convenience and potentially reducing background signal, thereby enhancing imaging effectiveness.^115^, ^116^

Second, comparison of outcomes from (pre)clinical NIRF tracer studies remains challenging due to heterogeneity in study design, imaging systems and reported endpoints. Some studies report quantitative TBR, while others focus on diagnostic metrics such as sensitivity and specificity, complicating cross‐study comparisons. Inconsistencies in tracer administration, timing and the use of control groups limit generalisability. Moreover, fluorescence signal detection and quantification are affected by biological factors (e.g., patient characteristics, tumour biology, perfusion) and technical factors (e.g., tissue optical properties, tracer administration and the performance of imaging systems). Together, these sources of variability complicate reliable comparison of outcomes across studies. A universal calibration tool for both open and laparoscopic tFGS is lacking and standardised protocols for dosage, timing and quantification are urgently needed.^6^, ^12^ Harmonised workflows and objective measures are essential to define clinically meaningful thresholds for malignant versus benign tissue differentiation.^95^

Further, attention should be drawn to the degree and heterogeneity of biomarker expression in different organs, cancer types and stages. Serum concentrations of the intended target do not accurately represent tumour biomarker expression, which makes serum concentrations for patient selection not reliable.

To enable broad implementation of tFGS, robust clinical evidence must first be established. Phase III/IV trials are needed not only to confirm diagnostic accuracy under routine clinical conditions but, more importantly, to demonstrate that tracer‐guided strategies lead to improved patient outcomes, such as reduced morbidity and enhanced survival. In parallel, early health technology assessments or cost–benefit analyses can help prioritise research directions that are most relevant for patients and society.^52^ Ultimately, reimbursement strategies should be investigated, where the financial added value of tFGS is determined by weighing production costs against demonstrated clinical benefit.

### 3.4 Future perspectives

#### 3.4.1 Innovations in TFGS

Extensive research has assessed diverse fluorescent tracers for tFGS. While clinical trials demonstrate its high potential in GI oncology, phase III/IV studies remain essential to confirm diagnostic accuracy and improved outcomes. To advance the field, several innovations are under investigation. Strategies to overcome limited intraoperative penetration include dual‐labelled tracers, photoacoustic imaging (PAI) and next‐generation NIR‐II fluorophores. Dual‐labelled tracers combine fluorophores with radionuclides or MRI agents to enable complementary multimodal imaging.^7^, ^20^, ^28^, ^32^, ^55^, ^56^, ^80^, ^118^, ^131^ PAI integrates NIR excitation with ultrasound detection, offering deeper, high‐resolution visualisation.^76^, ^83^, ^116^ A multimodal approach to overcome limitations related to optical signals, such as autofluorescence, depth and scattering, is deemed most promising to advance TFGS. NIR‐II fluorophores provide superior image quality compared with NIR‐I, but translation is hampered by complex chemistry, low tumour uptake, rapid clearance and lack of clinical systems.^31^, ^63^, ^86^, ^101^ Therefore, short‐term clinical implementation of NIR‐II fluorophores remains challenging at present, and current research efforts may be more effectively directed towards multimodal strategies with clearer translational potential. Beyond imaging, theranostic approaches such as photothermal, photodynamic and photoimmunotherapy aim to integrate diagnosis with targeted treatment.^41^, ^65^, ^66^, ^101^

#### 3.4.2 Success factors for clinical translation

To implement tFGS in routine GI oncology practice, several requirements must be met. Central to this transition is the generation of sufficient clinical evidence to satisfy regulatory agencies. While early‐phase studies have highlighted the potential of various tracers, current trials remain largely heterogeneous and exploratory, with limited follow‐up. To achieve approval, diagnostic accuracy must first be firmly established, followed by confirmatory phase III efficacy trials demonstrating that tFGS translates into improved surgical and oncological outcomes. The pivotal phase III trial with OTL38 in ovarian cancer (NCT03180307) illustrates this pathway: its design was agreed in advance with the US FDA under a Special Protocol Agreement, ensuring that positive results would be sufficient to support approval. Similar regulatory strategies will be required for tracers intended for GI oncology.

Evidence must also extend beyond diagnostic accuracy to include long‐term outcomes. Recent large‐scale studies on ICG and SGM‐101 suggest that widespread adoption without rigorous validation may not always improve surgical or oncological endpoints.^144^, ^145^ These findings underline the importance of robust, adequately powered trials with clinically relevant outcomes before broad implementation.

Cost effectiveness represents a second decisive factor. Demonstrating that the benefits of tFGS, such as reduced complications or shorter hospital stays, outweigh the costs of tracer production and imaging equipment will be essential to secure reimbursement and justify integration into standard care. While ICG gained traction partly due to its low cost and broad applicability and its reliance on passive distribution, more complex tracers such as OTL38 use active targeting mechanisms, which introduce additional complexity in molecular design and bio pharmacokinetics, accompanied by higher costs and logistical challenges. Early health technology assessments can guide whether investments in these agents are justified.

Finally, differences between regulatory frameworks must be considered. Whereas the US FDA offers pathways for accelerated approval under defined agreements, the European market is more fragmented, often requiring additional efforts to align standards across member states. For tracers to succeed, harmonisation of trial design, outcome measures and regulatory dialogue will be indispensable.

#### 3.4.3 Ongoing clinical trials

Ongoing trials reflect growing clinical interest in tFGS. SGM‐101 is most extensively studied, with trials across colorectal, rectal, pancreatic, brain and lung metastases (e.g., NCT03659448, NCT04642924, NCT05984810). EGFR‐targeted tracers (cetuximab, panitumumab) and ONM‐100 are also under investigation in oesophageal and peritoneal disease. Novel applications include RD‐Cy7 for ITGA6‐targeted imaging in HCC (NCT06204835) and CYTALUX (NCT07039526), which has not yet been studied in GI cancers. Furthermore, NCT06307548 explores intraoperative photodynamic therapy, a first in CRC. These studies will provide critical data on safety, efficacy and real‐world feasibility of tFGS across diverse settings. A detailed overview of these trials can be found in Table 2.

**TABLE 2:** Ongoing clinical trials tFGS to date (September 2025).

Clinical trial ID	Title	Tracer	Targeted organ	Status
NCT03659448	Performance of SGM‐101 for the delineation of primary and recurrent tumour and metastases in patients undergoing surgery for colorectal cancer	SGM‐101	Primary cT4 colon cancer or primary cT3/4 rectal cancer, recurrent crc or PM	Recruiting 17 June 2019
NCT05965817	Fluorescence‐guided resection of colorectal liver metastases using SGM‐101 and indocyanine GREEN (FOCUS GREEN)	SGM‐101 / ICG	Colorectal metastases	Recruiting 01 December 2023
NCT04755920	SGM‐101 in colorectal brain metastases	SGM‐101	Brain metastasis of colorectal origin	Recruiting 01 January 2024
NCT05984810	NIR‐fluorescence guided surgical resection of neoadjuvant treated localised pancreatic cancer using SGM‐101	SGM‐101	Neoadjuvant treated (borderline) resectable or locally advanced pancreatic cancer	Recruiting 18 March 2024
NCT04642924	SGM‐101 in locally advanced and recurrent rectal cancer (SGM‐LARRC)	SGM‐101	T3 with threatened CRM or T4 rectal cancer (locally advanced) or recurrent rectal cancer	Recruiting
NCT04737213	SGM‐101 in colorectal lung metastases	SGM‐101	Lung metastasis of colorectal origin	Completed
NCT06395337	Multimodal imaging in rectal cancer and pancreatic cancer	[^111^]In‐DOTA–ANTI‐CEA antibody	Clinical diagnosis of rectal cancer where a (beyond) TME resection is planned for OR Clinical suspicion of PDAC	Unknown
NCT03620292	Fluorescence image guided surgery in cholangiocarcinoma (COUGAR)	Bevacizumab–IRDye800CW	Clinical suspicion of PHCC	Unknown
NCT05497726	Fluorescent sentinel lymph node identification in colon carcinoma using intravenous bevacizumab–800CW (IBIZA‐2)	Bevacizumab–800CW	Pathologically confirmed and/or suspected cT1‐3N0‐2M0 colon carcinoma	Completed
NCT04638036	Fluorescence molecular endoscopy and molecular fluorescence‐guided surgery in locally advanced rectal cancer (TRACT‐II)	Cetuximab–IRDye800	Locally advanced rectal cancer	Completed
NCT04161560	Intraoperative fluorescence imaging in oesophagectomy of oesophageal cancer	Cetuximab–IRDye800	Oesophageal squamous cell carcinoma T1‐3N0‐1M0 patients	Unknown
NCT06168552	Fluorescent navigation technology in radical resection of pancreatic cancer (CISPD‐6)	Anti‐EGFR‐IR800CW	Radical surgical resection of pancreatic cancer	Not yet recruiting
NCT05518071	FLUOPANC‐trial—fluorescence‐guided surgery of pancreatic and bileduct tumours using cRGD–ZW800‐1	cRGD–ZW800‐1	Histologically proven pancreatic carcinoma with or without neoadjuvant treatment	Completed
NCT06307548	Fluorescence image guided surgery followed by intraoperative photodynamic therapy for improving local tumour control in patients with locally advanced or recurrent colorectal cancer	Aminolevulinic acid	Locally advanced or recurrent colorectal cancer	Recruiting 30 July 2024
NCT06511037	PhI pilot study pafolacianine inject for intraoperative imaging on outcomes of gi cancer peritoneal carcinomatosis	Pafolacianine (CYTALUX)	Gastrointestinal cancers and peritoneal carcinomatosis during cytoreductive surgery	Recruiting 13 November 2024
NCT07039526	Single dose investigator initiated pilot study to investigate CYTALUX (pafolacianine) for intraoperative detection of malignant tissue in subjects undergoing surgical resection for cancer	CYTALUX (pafolacianine)	Primary diagnosis of GI, fore gut, pancreatic, hepatobiliary, oesophageal and gyn malignancies planned for HIPEC/debulking	Not yet recruiting
NCT04950166	A study to evaluate ONM100, an intraoperative fluor imaging agent for the detection of peri mets	ONM‐100 (pegsitacianine)	Confirmed metastatic disease of peritoneal origin	Completed
NCT05047510	GPC3 targeted fluorescence image guided surgery of hepatocellular carcinoma	Anti‐GPC3–IRDye800CW	HCC	Recruiting 10 September 2021
NCT06204835	ITGA6 targeting NIR‐II fluorescence image guided surgery	RD‐Cy7 fluorophore	HCC with highly expressed ITGA6	Recruiting 07 January 2024

## 4 CONCLUSION

tFGS holds substantial promise to transform intraoperative decision‐making and improve oncological outcomes in GI cancer. This systematic review demonstrates encouraging data for a broad range of molecular targets and fluorophores, with several tracers already advancing into phase II/III trials. However, widespread clinical translation remains constrained by methodological heterogeneity, logistic hurdles and the lack of standardised evaluation frameworks. Looking ahead, innovations such as multimodal tracers, NIR‐II fluorophores and theranostic agents may further enhance the precision and therapeutic potential of tFGS. With interdisciplinary collaboration and patient‐centred research, the field is moving from promise to practice, bringing physicians one step closer to personalised oncologic precision surgery.

## AUTHOR CONTRIBUTIONS

*Data curation, formal analysis and writing – original draft*: Aaya Darai. *Data curation, formal analysis and writing – original draft*: Evie H. M. Graus. *Data curation and writing – review and editing*: Femke J. A. van der Stroom. *Conceptualisation, supervision and writing – review and editing*: Mark Rijpkema. *Supervision and writing – review and editing*: Alexander Vahrmeijer. *Supervision and writing – review and editing*: Denise E. Hilling. *Conceptualisation, supervision and writing – review and editing*: Johannes H. W. De Wilt. *Conceptualisation, supervision and writing – review and editing*: Merlijn Hutteman.

## CONFLICT OF INTEREST STATEMENT

The authors declare no conflicts of interest.

## ETHICS STATEMENT

Ethical approval was not required for this systematic review, as all data were derived from previously published studies and no new studies involving human participants or animals were conducted.

## Supporting information



Supporting Information

Supporting Information

## Data Availability

The data supporting the findings of this systematic review are available within the article and its supporting information. Extracted data tables are provided in supporting information.
