# Supplementary material for: Tumour‐targeted fluorescence‐guided surgery in gastrointestinal cancer: A systematic review of preclinical and clinical research
Source: Clin Transl Med. 2026 Mar 22;16(3):e70615. doi: 10.1002/ctm2.70615 (PMC13093331; doi:10.1002/ctm2.70615)
Supplement: Supplementary file 1 — Supporting Information [file CTM2-16-e70615-s001.docx]

**Table 1: Preclinical research CEA targeting tracers**

| **Fluorescent Agent** | **Fluorophore** | **Excision - Emission wavelength** | **Studysize Target organ(s)** | **Study outcome** |
| --- | --- | --- | --- | --- |
| Anti-CEA M5A-IR800-SW | IRDye-800CW | 778nm - 794nm | N= 6, gastric cancer PDOX mouse model, N = 5, control | TBR = 6.25 primary gastric cancer PDOX, TBR = 0.42 control  TBR = 13.52 abdominal wall metas, TBR = 3.19 control [12] |
|  |  |  | N = 9, CRC primary tumours subcutaneous mouse model, CRC primary and liver metastasis orthotopic mouse model | TBR = 3.44 – 12.75 (subcutaneous model), highest TBR observed for the 75 μg dose at 96 h [13] |
|  |  |  | N = 5 KG8 gastric cancer PDOX models, control N =4  N = 6 KG10 gastric cancer, control N=5 | In the *KG8* PDOX models, the TBR for M5A-IR800 was 5.85 (SE ± 1.64) compared with IgG-IR800 at 0.70 (SE ± 0.17). The *K10* PDOX models had a TBR of 3.71 (SE ± 0.73) for M5A-IR800 compared with 0.66 (SE ± 0.12) for IgG-IR800 [14] |
|  |  |  | N = 2 x 14 = 28 gastric cancer PDOX mouse model & control | 59.5% of LNs contained metastatic gastric cancer cells. Sign difference in mean fluorescence signal for cancer positive LNs vs. cancer negative LNs(0.431 vs 0.105) [15] |
|  |  |  | N = 34 gastric cancer or PM (N = 26 primary tumour, N = 8 PM, n = 2 carcinomatosis, n = 19 positive LN) | TBR = 4.28 gastric cancer vs. TBR = 0.82 control [16] |
|  |  |  | N = 9, CRC liver metastases orthotopic mouse model | Mean (SD) Tumour 1.644(+/-0.807), Liver 0.251(+/-0.118) Tumour-to-liver ratio = 6.52 (+/-2.710) (72 h post administration)  Co-localized with luciferase [17] |
| anti-CEA-IRDye800CW | IRDye-800CW | 778 nm – 794 nm | N = 3, CRC orthotopic mouse model | Strong fluorescence observed, with decreasing intensity over time. High specificity confirmed; liver and kidney also showed signal due to metabolism. No fluorescence detected in blood after 48 h. [18] |
| 111In-RDC018 / 111In-IMP288 | IRDye800CW | 774 nm-  789 nm | N = 2 x 30 = 60 PDOX CRC mouse model (¹¹¹In-IMP-288 & ¹¹¹In-RDC018)  N = 2 x 5 = 10 PDOX CRC mouse model (¹¹¹In-RDC018 vs. dual-labeled hMN-14) | 111In-RDC018 showed specific tumour targeting in pretargeted CEA-positive tumours (21.9 ± 4.5 and 10.0 ± 4.7% injected activity per gram (mean ± SD %IA/g), at 2 and 24 hours post-injection (p.i.), respectively) and a biodistribution similar to 111In-IMP288 [19] |
| 111In-DTPA-MN-14-IRDye 800CW | IRDye-800CW | 773 nm-792 nm | N = 5 x 5 = 25, Subcutaneous CRC mouse model  N = 2 x 8 = 16, Subcutaneous CRC mouse model vs. control  N = 4 x 5 = 20, Subcutaneous CRC mouse model  N = 2 x 4 = 8, CRC orthotopic mouse model (dual-labeled vs. control preoperative SPECT & FGS) | Tumour uptake: 24.0 ± 2.3 %ID/g at 72 h p.i.  TBR (fluorescence): 4.7 ± 0.5  Fluorescence signal corresponded to radiotracer signal [20] |
| hM5A-IR800 | IRDye800CW | 778 nm – 800 nm | N = 6 x 3 = 18, PDAC orthotopic PDAC mouse model (6, 12, 24, 48, 72, and 96 hours after injection) | There was a clear tumour signal with a tumour-to-TBR > 5 at all time points, with high contrast (TBR of 16.6) at 48 hours. [22] |
|  |  |  | N = 2 x 5 = 10, PDAC PDOX mouse model & control | The average TBR was 3.5 [23] |
| Anti-CEA nanobody–IRDye800CW | IRDye800CW | 778 nm – 800 nm | N = 2 x 5 = 10, orthotopic PDAC mouse model & control group | The signal was present as early as 15 minutes after injection and was robust at 1 to 3 hours after injection with a TBR of 2.66  Control nanobody did not show any tumour-specific signal [24] |
|  |  |  | N = 10, subcutaneous PDAC mouse model, N = 4, PDAC PDOX mouse model | Probe rapidly localized to the pancreatic cancer tumours within an hour and had a tumour-to-background ratio of 2.0 by 3 h [25] |
|  |  |  | CRC PDOX mouse model  & control | Maximal fluorescence intensity was observed within 15 min-3 h for all three doses with TBR values ranging from 1.3 to 2.3. In the patient-derived model of colon cancer, fluorescence was detectable with a TBR of 4.6 at 3 h. [26] |
| anti-CEA M5A-sidewinder | IRDye800CW | 778 nm – 800 nm | N = 2 x 4 = 8 , subcutaneous CRC mouse model (PET/MR/NIR fluorescence & blood clearance) | PET scans showed higher activity for the liver than the tumour, compared to the NIR fluorescence imaging. It quantifies the expected difference due to the sensitivity and depth of penetration between the 2 modalities. [27] |
|  |  |  | N = 2 x 4 = 8 , subcutaneous CRC mouse model (PET/MR/NIR fluorescence & blood clearance) | PEGylated linker (M5A-SW-IR800): TBR = 10.1 ± 1.6 Conventional (non-PEGylated) linker (M5A-IR800):  TBR = 5.5 ± 0.6 [28] |
| ssSM3E/800CW | IRDye800CW | 773 nm / 792 nm | N = 3, CRC PDOX mouse model  N = 4, PDAC PDOX mouse model | TBR = 2.55 ± 0.7 (CRC) TBR = 2.37 ± 0.4 (PDAC)[29] |
| 2D5-IRDye800CW | IDRye800CW | 789nm-776nm NIR‑II tail extending to 1600 nm | N = 15 subcutaneous CRC  N = 15 orthotopic CRC  N = 10 peritoneal metastases | Tumour‑to‑background ratio (TBR) was significantly higher in NIR‑II compared to NIR‑I (2.55 ± 0.38 vs 1.94 ± 0.20, P < 0.0001) across 30 models [30] |
| 111In-DTPA-labetuzumab-IRDye800CW | IRDye-800CW | 778nm - 794nm | N = 4 x 7 = 28, pulmonary micro metastases of CRC origin PDOX mouse model | TBR = 17.2 ± 5.4 and 16.5 ± 4.4 at weeks 3 and 4, respectively [31] |
| anti-CEA-Alexa 488 / anti-CEA-Alexa 555 / anti-CEA-Alexa 647 | Alexa 488 / Alexa 555 / Alexa 647 | Alexa 488:  496 nm – 519 nm  Alexa 555:  555 nm – 565 nm  Alexa 647:  650 nm – 665 nm | Metastatic PDAC orthotopic mouse model | The simultaneous use of different fluorophores (Alexa 488 and Alexa 555) conjugated to antibodies brightened the fluorescence signal, enhancing detection of sub-millimeter lesions without compromising background illumination. [32] |
|  |  |  | N = 3 x 8 = 24 PDAC orthotopic mouse model (bright-light surgery vs. FGS vs. GFS-Ultraviolet) | Complete resection was achieved in 92 % of mice in the FGS group compared to 45.5 % in the BLS group. [33] |
|  |  |  | N= 46, PDAC PDOX mouse model  (N = 24 FGS & N = 22 bright-light surgery) | the FGLS group had less pancreatic tumour volume than the BLLS group (5.75 mm2 vs 28.43 mm2, respectively; and lower tumour weight (21.1 mg vs 174.4 mg, respectively. FGLS compared to BLLS also decreased local recurrence (50% vs 80%) and distant recurrence (70% vs 95%). [34] |
|  |  |  | N = 3 PDAC orthotopic mouse model  N = 10 orthotopic carcinomatosis mouse model | 105 of lesions were detected under NIRF imaging (cumulative sensitivity 96.3%), whereas only 44 were picked up under bright light (cumulative sensitivity 40.4%; p<0.001) [35] |
|  |  |  | PDOX colon cancer mice model  N=unknown | anti-CEA antibody administered as a single intravenous dose 24 hours before laparotomy selectively labeled cancer cells in the colon cancer PDOX, tumour fluorescence was present for at least 1 week. [36] |
|  |  |  | PDOX colon cancer mice model  N= 22 FGS, N= 21 mice BS | CEA-expressing tumours labeled with chimeric CEA antibody provided a brighter fluorescence signal on frozen human tumour tissues (*P* = 0.046) The R0 resection rate increased from 86% to 96% with FGS compared to BLS. [37] |
| (mAb) 6G5j-IR800CW  6G5j-IR700DX/ | IR800CW  IR700DX & ICG | IRDYe800CW: 774nm-789nm | N = 10 mice or colon cancer/metastasis  N=10 mice | Optimal tumour visualization occurred at 48 h after administration of 50 µg. tumour‑to‑liver ratio (TLR) ~3.17 ± 0.45 [21, 38]  tumour fluorescing at ~700 nm (6G5j-IR700DX) vs. perfused liver segment fluorescing at ~800 nm (ICG). Mean tumour-to-liver ratio (TLR) for CM2 group ~4.40 ± 0.96, Liver5 ~3.38 ± 1.25 |
| 25I-MAb 35A7-indocyanine | indocyanine green | 649 nm- 670 nm | N = 6 x 5 = 30, (n = 25, Peritoneal carcinomatosis CRC orthotopic mouse model & n = 5 control group) | Detection of very small tumour nodules (<1 mg / <1 mm) with 78% sensitivity  Overall performance: Sensitivity: 90.7% Specificity: 97.2% PPV: 94.7% NPV: 94.9% [39] |
| CEA(Ab)-MSNs-ICG-Pt | indocyanine green | 785 nm / 813 nm | N = 4 x 5 = 20, CRC PDOX mouse model (control, Pt, MSNs-ICG-Pt, and CEA (Ab)-MSNs-ICG-Pt) | Strong tumour-specific accumulation in vivo with high fluorescence signal in subcutaneous CRC tumours and a labeling ratio of 92.3% in vitro. The CEA-targeted nanoparticle significantly inhibited tumour growth compared to controls and showed high biocompatibility and safety in mice [40] |
| anti-CEA-DyLight750 /anti-CEA-DyLight650 | DyLight750/ DyLight650 | DyLight650: 652 nm – 672 nm  DyLight750:  752 nm – 776 nm | N = 60 nude mice for serum concentration  N = 4 x 5 nude mice for tissue biodistribution  N = 2 x 8, PDAC  PEGylated and non PEGylated (DyLight750 & Dylight650) orthotopic mouse model | The PEGylated complexes labeled the tumour significantly brighter and for longer periods. The PEGylated dyes conjugated to anti-CEA labeled the tumour significantly brighter. [41] |
|  |  |  | N = 3 x 5 = 15 CRC liver metastasis orthotopic mouse model (bright-light surgery vs. FGS with GFP vs.  FGS with anti-CEA-DyLight650 | Mice that underwent FGS had a significantly smaller area of residual tumour, longer overall survival and disease-free survival (P < 0.001). [42] |
| SGM-101 | BM-104 | 660/680 nm – 689/725 nm | N = 4 x 5 = 20, CRC, PM, liver metastases orthotopic mouse models for biodistribution  N = 8, PM orthotopic mouse model | TBR = 3.5 ± 0.6 (SGM-101)  TBR = 1.2 ± 0.5 (BM-104, control)  [43] |
|  |  |  | N = 184 rats (healthy)  N = 6 beagle dogs (healthy)  N = 42 human frozen tissues  Transgenic mice that express human CEA | Absence of significant adverse effects of both SGM-101 and BM-104 at doses well above the anticipated maximal human exposure  [44] |
|  |  |  | N = 5 gastric cancer subcutaneous xenograft model + n = 2 control  N = 9 peritoneal subcutaneous xenograft mouse model + n = 3 control | Signal intensity correlates with CEA exprssion. Clear visualization of gastric tumours and peritoenal lesions [45] |
| [111In]In-DTPA-SGM-101 | BM-104 |  | N = 4 x 5 = 20, Subcutaneous CRC mouse model (3, 10, 30, or 100 μg)  N = 3 x 5 = 15, Subcutaneous CRC mouse model (24, 48, or 72 hours)  N = 6, orthotopic CRC mouse model (SPECT and FGS)  N = 43, peritoneal tumour specimen | TBR = 5.8 ± 1.1 (30 μg)  MFI = 1371 (peritoneal tumour specimen) & MFI = 293 (normal tissue) (P < 0.001) [46] |
| NbCEA5-ZW800F /NbCEA5-ZW800-1 | ZW800-F/ ZW800-1 | 685/754nm – 772nm  772nm - 788nm | N = 11 subcutaneous PDAC mouse model N= 2, PDAC orthotopic mouse model N=3, control | Superior mean fluorescence intensities for NbCEA5-ZW800-1 vs NbCEA5-ZW800F.PDAC models, NbCEA5-ZW800-1 accumulated in tumour mean in vivo TBR of 2.4 (SD = 0.23). [47] |
| CEA-targeted nanoparticle | Cy5.5 | 675 nm – 694 nm | N = 10, subcutaneous CRC mouse model N = 2 x 3, (PDOX CRC mouse model & control group) | In PDOX models, tumour fluorescence was clearly visible with a TBR ratio (TBR) of 3.3 ± 0.4 at 24 h post-injection. [49] |

**Table 2: Preclinical research VEGF targeting tracers**

| **Fluorescent Agent** | **Fluorophore** | **Excision -Emission wavelength** | **Studysize**  **Target organ(s)** | **Study outcome** |
| --- | --- | --- | --- | --- |
| BevacizumabIRDye 800CW | IRDye 800CW | 774 nm – 789 nm | N = 10 orthotopic mouse models (A2780luc1, SKOV3‑luc1) | 6 days post injection (TBR) was 1.93 ± 0.40 [6] |
|  | IRDye 800CW | 774 nm – 789 nm | N = 11, peritoneal carcinomatosis of ovarian origin PDOX mouse model | Amount of unresected tumour residues in mice was significantly lower with Angiostamp800 or Bevacizumab-IRDye 800CW than with ICG higher specificity and lower false positive (3.5%)  [145] |

**Table 3: Preclinical research EGFR targeting tracers**

| **Fluorescent Agent** | **Fluorophore** | **Excision -Emission wavelength** | **Studysize**  **Target organ(s)** | **Study outcome** |
| --- | --- | --- | --- | --- |
| Cetuximab / trastuzumab-Alexa/IRDYe QC-1 | Alexa Fluor 700 (AF700), IRDye QC-1 (QC-1) | AF700: 702 nm – 723 nm  QC-1: 788 nm - none  788 nm - none | PDAC PDOX mouse model | The dual-activatable probe approach is shown to enhance the specificity. [60] |
| Cetuximab-VivoTag 680 | VivoTag 680 | 673 nm – 691 nm | N= 15, prostate, colon, lung cancer orthotopic mouse models | Detection limit: 3-5 mm [61] |
| cetuximab-IR800 | IRDye-800CW | 808-1000nm | Orthotopic ESCC mouse model | subcutaneous tumours, the TBR increased from 1.58 ± 0.09 at 6 h to 3.95 ± 0.10 at 120 h**.** NIR‑II guided surgery (808/1000 nm) produced a significantly higher TBR (2.11 ± 0.46) compared with NIR‑I imaging (760/845 nm) (1.58 ± 0.31) [62] |
|  | Cetux-Alexa-647 / IRDye 800CW | Cetux-Alexa-647:  640 nm – 690 nm  IRDye 800CW:  785 nm – 808 nm | N =17, orthotopic PDAC mouse model (n = 10 AsPC-1 & n = 7 MIA PaCa-2) (Cetux-Alexa-647)  N = 5, PDAC PDOX mouse model (IRDye 800CW) | Fluorescence guided surgery performed 24 h after injection delineated the primary tumour, surgical scar mass and metastases; only viable tumour tissue fluoresced, and metastases as small as ~1 mm were detected while healthy tissues showed no signal. [63] |
| PEG-GNR-Cy5.5-anti-EGFR-antibody | Cy5.5 | 678 nm – 695 nm | N = 2 x 10 = 20, ESC PDOX mouse model (Intratumoural & Intravenous gold nanorods) | TBR: 3.2 at 12h. Used for dual-mode imaging + photothermal therapy. Reduction in tumour size of 72% post-therapy [64] |
| H2a-4T@Cetuximab | H2a-4T | 785 nm – 1033 nm | N = 2 x 3 = 6, orthotopic CRC mouse model (H2a-4T & H2a-4T@FBS) | TBR: 4.9 at 24h. Demonstrated effective tumour ablation in vivo with >80% tumour volume reduction compared to controls [65] |
| IRDye800CW-nimotuzumaB | IRDye800CW | 774 nm – 789 nm | A-431 (epidermoid carcinoma), DLD-1 (CRC), and MDA-MB-435 (negative control) PDOX mouse models | In EGFR-positive xenografts, IRDye800CW-nimotuzumab showed more than 2-fold higher uptake in tumours compared to IRDye800CW-cetuximab. In addition, liver uptake of IRDye800CW-nimotuzumab was two-fold lower than cetuximab. [66] |
| Tras-AA-Cy NH2 | CY dye | 675 nm – 720 nm | PDOX gastric cancer mouse model | TBR: 6.3 at 6h post-activation. Activatable probe improved contrast. 100% lesion detection in peritoneal metastases >1 mm [67] |
| Panitumumab-IRDye800CW | IRDye-800CW | 775-795nm | N = 3 x 10 = 30, orthotopic CRC mouse model (LS174T, Colo205, SW948) | Average TBRs were 6.00 vs. 2.60 for LS174T, 5.78 vs 2.52 for Colo205 and 4.31 vs 1.70 for SW948 [68] |
| Rybrevant-IRDye800CW | IRDye800CW | 778 nm – 778 nm | N = 2 x 3, CRD PDOX mouse lymph node metastases N = 25 CRC surgical specimen | Dual target probe (EFGR & c-Met) TBR = 2 (6h post injection) TBR = 2.55 vs. TBR = 1.94 (NIR-II vs. NIR-I). Fluorescence intensity of tumour tissue is significantly higher than normal intestinal mucosal tissue [69] |

**Table 4: Preclinical research Integrin αvβ targeting tracers**

| **Fluorescent Agent** | **Fluorophore** | **Excision -Emission wavelength** | **Studysize**  **Target organ(s)** | **Study outcome** |
| --- | --- | --- | --- | --- |
| IntegriSense680 | VivoTag®-S680 | 680 nm - 700 nm | Orthotopic CRC mouse model | Statistically significant discrimination of tumour and healthy tissue was achieved based on their specific fluorescent signal patterns and by absolute quantification of the fluorescent signal intensities. [72] |
|  |  |  | N = 7, syngeneic CRC rat model  (liver and peritoneal metastases) | Colorectal metastases had a minimal two-fold higher NIR fluorescence signal than healthy liver tissue and other abdominal organs (p < 0.001).  [73] |
|  |  |  | Orthotopic PDAC mouse model | Tracer enabled early and accurate in vivo detection of pancreatic cancer [79] |
| bioactivated in vivo assembly (BIVA) probe (named M1) | imaging motif (IR783) | 780 - 845 nm | N = 3, orthotopic PDAC mouse model (biodistribution)  N = 5, orthotopic PDAC mouse model (imaging)  N = 8, orthotopic PDAC mouse model (tumour vs. spleen quantification) | TBR = 5.12 (±0.35) at 6 h post-injection. Tumour clearly delineated; minimal background [74] |
| cRGD-800CW-TCO | IRDye800CW | 774 nm – 789 nm | N = 3, subcutaneous PDAC mouse model  N = 14, orthotopic PDAC mouse model | Highly sensitive delineation of tumour burden was achieved during FGS in all mice Highest TBR = 1.8 [75] |
| cRGD-ZW800-1 | ZW800-1 | 770 - 790 nm | n = 4, orthotopic CRC mouse model & n = 3 control group  N = 4 x 3 = 12 orthotopic CRC mouse model (biodistribution)  N = 5 orthotopic mouse models, (n = 2 oral, n = 2 breast, n = 1) | TBR at 4 h 1.8 ± 0.4, (CRC) [76] |
|  |  |  | N = 3 x 3 = 9, subcutaneous CRC mouse model (cRGD-ZW800-1, cRAD-ZW800-1, ZW800-1)  N = 6, orthotopic CRC mouse model | A significantly higher SBR ratio was observed in mice injected with cRGD-ZW800-1 (2.42 ± 0.77) compared with mice injected with cRAD-ZW800-1 or ZW800-1 alone (1.21 ± 0.19 and 1.34 ± 0.19, respectively) [78] |
|  |  |  | Metastatic to liver and long of melanoma origin PDOX mouse models | TBR of 17.2 is achieved at 4 h after injection of ZW800-1 conjugated to cRGD compared to ratios of 5.1 with IRDye800-CW and 2.7 with Cy5.5 [80] |
| SPIO@Liposome-ICG-RGD | ICG | 780 nm – 830 nm | N = 5, orthotopic HCC mouse model  N = 5, orthotopic intrahepatic metastases mouse model  N = 10, subcutaneous HCC mouse model | Contrast-to-noise ratio obtained from MRI was 31.9 ± 25.4Maximum TBR of NIRF imaging was 2.5 ± 0.3 at  72 h post-injection for effectively capturing miniscule tumour lesions (0.6 ± 0.3 mm) intraoperatively. [81] |
| RAFT-c(RGDfK)4-Alexa Fluor 700 | alexa fluor 700 | 702 nm – 723 nm | N = 15, orthotopic peritoneal carcinomatosis mouse model | Under normal light, only 50.6(2.3) % of the nodules that were visible under NIR light were detected.  The duration of surgery was reduced from 19.5(3.3) min under normal light to 14.0(2.6) min when NIR light was used (P = 0.025).  The sensitivity of the NIR system allowed the detection of nodules containing as few as 227 tumour cells. [77] |
| IR820-E[c(RGDfK)] | IR820-NHS ester | 822 nm – 835 nm | Orthotopic HCC mouse model | Preoperative tumour localization, intraoperative tumour boundaries delineation, and tumour excision, and postoperative negative margin assessment were successfully achieved during irregular hepatectomy. [82] |
| R01-MG-IRDye800 | IRDye800CW | 774 nm – 789 nm | N = 20 (n = 10, subcutaneous PDAC mouse model & n = 10 control)  N = 8, xenograft PDAC mouse model  N = 10, transgenic PDAC mouse model  N = 3, control (toxicity) | Tumour-specific uptake of R01-MG-IRDye800 was shown compared with IRDye800 alone (TBR 2.7 vs. 0.86). The fluorescent signal in tumours of transgenic mice was significantly higher, TBR of 3.6 ± 0.94, compared with the normal pancreas of wild-type controls, TBR of 1.0 ± 0.17 (P < 0.001). [83] |
| 5-FAM-X-PEG 28 -A20FMDV2-K16R-PEG 28 & IRDye800-PEG 28 -A20FMDV2-K16R-PEG 28 | FAM-X & IRDye800CW | 774 nm – 789 nm | N = 12, subcutaneous PDAC tumour model  N = 7, orthotopic PDAC mouse model | In vivo images and ex vivo biodistribution confirmed uptake and retention of 3 in the αvβ6 positive subcutaneous and orthotopic tumours, with negligible uptake in the αvβ6-negative tumour [84] |
| APP-Ag2S-RGD | Ag2S | 1200 nm | N = 15, orthotopic peritoneal metastasis mouse model | Superior TNR is achieved and tiny tumour metastatic foci as small as about 0.2 mm in diameter could be facilely eliminated under NIR-II fluorescent imaging guidance. TBR=13.60 tumour vs 3.36 normal [85] |
| DCNPs@Si-omSi-RGD | Nd-based nanocrystals | 808 nm – 1064 nm | N = 2 x 5 = 10, Subcutaneous liver mouse model (bright-light surgery vs. NIRII FGS) | Peak SBR = 15.3 (48 h post injection)  No long-term toxic side effects with significant prolonging survival rate [86] |
| Gd-Cy7-PTP/RGD | Cy7 | 756 nm – 779 nm | Subcutaneous and orthotopic PDAC mouse models | PTP/RGD peptides greatly enhanced the specificity of probe and increased its binding efficiency to PDAC Maximum TBR = 9.46 (4 h post injection) [87] |
| RVLu@ICG | ICG | 808 nm - ~ 1000 nm | CRC mouse models | TBR = 6.50 Radio-immunotherapy antitumour effect  NIR-II: clear differentiation of tumour boundaries [88] |
| MLRs | hemicyanine | 660 nm – 710 nm | Orthotopic gastric cancer models | MLR1 distinguishes macro/micrometastatic LNs from benign LNs . MLR1 differentiates cancerous tissues and metastatic LNs from normal tissues and benign LNs within 1 h [89] |
| Angiostamp™ 800 | Angiostamp fluorophore | 774 nm – 797 nm | N = 13, peritoneal carcinomatosis of ovarian origin PDOX mouse model | Amount of unresected tumour residues in mice was significantly lower with Angiostamp800 or Bevacizumab-IRDye 800CW than with ICG higher specificity and lower false positive (1.4%)  [145] |

**Table 5: Preclinical research 5-ALA targeting tracer**

| **Fluorescent Agent** | **Fluorophore** | **Excision -Emission wavelength** | **Studysize Target organ(s)** | **Study outcome** |
| --- | --- | --- | --- | --- |
| δ-aminolevulinic acid | **-** | 380 – 440 nm | N = 2 x 6 = 12 Peritoneal carcinosis induced rats (CRC cells) (270 vs. 340 g) | all tumours (100%) were fluorescence positive, whereas in the i.v. group only 32 of the tumours (28%) showed the typical red fluorescence. In the i.p. group, 30 additional tumours were detected by fluorescence excitation (21%), as compared with eight additional tumours in the i.v. group (7%). [91] |

**Table 6: Preclinical research folate receptor targeting tracers**

| **Fluorescent Agent** | **Fluorophore** | **Excision -Emission wavelength** | **StudysizeTarget organ(s)** | **Study outcome** |
| --- | --- | --- | --- | --- |
| IRFEP FA DOTA Gd (IFDG | IRFEP (NIR‑II fluorophore) | 545 nm - 808 nm | N =12, orthotopic HCC tumour model | NIR II signals peaked ≈24 h after injection and persisted for ≥48 h. [100] |
| ZGC FA | - | persistent luminescence at 695 nm | Orthotopic CRC mouse model | SNR = 23. 0 (in-vivo) Complete tumour resection was achieved under PersL guidance, with only 2.3% of healthy tissue removed. [101] |

**Table 7: Preclinical research mucin targeting tracers**

| **Fluorescent Agent** | **Fluorophore** | **Excision -Emission wavelength** | **Study size - Target organ(s)** | **Study outcome** |
| --- | --- | --- | --- | --- |
| P-(EPPT1)-FITC / P-(EPPT1)-IR783 | FITC / IR-783 | 488 - 550 nm – 515 - 570 nm /  780 - 790 nm – 800 - 820 nm | N = 7 human CRC tissue specimens  N = 65, CRC PDOX mouse model | Three times higher fluorescence intensity compared to control group [105] |
| MUC4-IR800 | IRDye-800CW | 774 nm – 789 nm | N = 2, CRC subcutaneous mouse model  N = 3, liver subcutaneous mouse model  N =  3, primary CRC PDOX mouse model  N = 2 liver metastases PDOX mouse model | TBR = 2.17, primary CRC  TBR = 2.56, liver metastases of CRC origin [106] |
| Anti-MUC4-IRDye800CW | IRDye800CW | 774 nm – 789 nm | N = 2 x 5, PDAC PDOX mouse model  N = 2 x 4, peritoneal carcinomatosis PDOX mouse model | TBR = 2.27 - 2.42 TLR = 7.74 – 9.05 [108] |
| AR9.6-IRDye800 | IRDye-800CW | 774 nm – 789 nm | N = 3, subcutaneous PDAC mouse model | TBR > 3, peak TBR = 6.95 [109] |
|  |  |  | N = 6 x 3 = 18, PDOX PDAC mouse model (6, 12, 24, 48, 72 & 96 hours post-injection) | AR9.6-IRDye800 exhibited superior fluorescence enhancement of tumours and lower signal in critical background organs in comparison to a nonspecific IgG control [110] |
| MUC5AC-IR800 | IRDye-800CW | 774 nm – 789 nm | N = 6, PDAC subcutaneous mouse model  N = 6, PDAC PDOX mouse model | TBR = 2.46, subcutaneous model  TBR = 4.35, PDAC model (72 h, 75 µg) [112] |
|  |  |  | N = 4, PDAC subcutaneous mouse model  N = 3, liver metastases PDOX mouse model  N = 1 human PDAC liver metastases specimen | TBR = 7.034, subcutaneous model  TBR = 1.787, liver metastases model [111] |

**Table 8: Preclinical research EpCAM targeting tracers**

| **Fluorescent Agent** | **Fluorophore** | **Excision -Emission wavelength** | **Study size - Target organ(s)** | **Study outcome** |
| --- | --- | --- | --- | --- |
| Anti-EpCAM/800CW | IRDye-800CW | 785 nm – 808 nm | N = 2 x 3, subcutaneous CRC mouse model (323/A3-800CW & control)  N = 2 x 3, CRC PDOX mouse model (323/A3-800CW & control)  N = 2 x 3, Breast carcinoma PDOX mouse model (323/A3-800CW & control)  N = 2 x 3, Head-and-neck PDOX mouse model (323/A3-800CW & control)  N = 3, peritonitis carcinomatoses PDOX mouse model (323/A3-800CW) | TBR = 13.5 (72h), CRC vs TBR = 1.8, control  TBR = 6.7 (72h), breast carcinoma vs TBR = 1.9, control  TBR = 4.9 (72h), head-and-neck cancer vs TBR = 1.6, control  Accuracy = 98% (sens = 93% & spec = 92%), peritonitis carcinomatoses  [113] |
| EpCAM-F800 | IRDye-800CW | 773 nm – 792 nm | N = 2 x 6 (52 µg & control), subcutaneous CRC mouse model  N = 6 x 3 (3.3 µg, 13 µg, 52 µg, 260 µg, 1040 µg), subcutaneous CRC mouse model  N = 4 x 2 (1h, 4h, 8h, 24 h), subcutaneous CRC mouse model  N = 2 x 3 (24h & 72h – 150 µg), CRC PDOX mouse model  N = 2 x 6 (24h & 72h – 52 µg), CRC PDOX mouse model | TBR = 11.5 (24h) & TBR = 8.9 (72h), subcutaneous CRC mouse model  TBR = 6.8, CRC vs. TBR = 2.1, control (24h)  TBR = 10.0, CRC vs. TBR = 3.0, control (72h)  [114] |
| Ac2-800CW  Ec4.1 -800CW | IRDye-800CW | 780 -785 nm – 805 - 820 nm | N = 6, subcutaneous CRC mouse model  N = 12, CRC PDOX mouse model | TBR = 4.2 (Ac2) & TBR = 5.3 (Ec4.1), CRC PDOX mouse model  TBR = 2.6 (Ac2) & TBR = 3.1 (Ec4.1), CRC PDOX mouse model [115] |
| ICG-CuS-Gd@BSA-EpCAM NPs | ICG | 745 nm - 840 nm | N = 3 x 5 = 15, Liver PDOX mouse model (ICG, ICG-CuS-Gd@BSA, ICG-CuS-Gd@BSA-EpCAM), | TBR > 3  Peak fluorescence intensity at 12h (ICG group)  Peak fluorescence intensity at 24h (, ICG-CuS-Gd@BSA, ICG-CuS-Gd@BSA-EpCAM [116] |

**Table 9: Preclinical research uPAR targeting tracers**

| **Fluorescent Agent** | **Fluorophore** | **Excision -Emission wavelength** | **Study size - Target organ(s)** | **Study outcome** |
| --- | --- | --- | --- | --- |
| hybrid ATN-658 | ZW800-1 | 773 nm – 790 nm | N = 3 x 6 = 18, subcutaneous CRC mouse model (CRC & control + 50 µg, 100 µ, 150 µg)  N = 2 x 3 = 6, CRC PDOX mouse model (CRC & control, 75 μg/0.5 nmol)  Peritoneal carcinomatosis PDOX model | NIR: TBR = 3.9, subcutaneous CRC model  SPECT: TBR = 3.4 (6h), TBR = 4.2 (24h), TBR = 3.1 (48h), TBR = 4.0 (72h), subcutaneous CRC model  TBR = 5.0 (72h) CRC PDOX model vs. TBR = 1.3 (control)  Visualization of small lesions 1-2 mm possible [117] |
| ICG-Glu-Glu-AE105 | ICG | 780 nm – 830 nm | N = 5, PDAC PDOX mouse model  N = 8, PDAC metastases PDOX mouse model | TBR = 3.5, primary tumour, TBR = 3.4, metastatic tumour  50% of mice additional metastasis only found with tFGS, 14% additional metastases removed under NIR light vs. white light imaging [118] |
| UlgG-800F/uFab2-800F/uFab-800F | IRDye800CW | 785 nm – 808 nm | Subcutaneous CRC mouse model, PDAC PDOX mouse model, Head-and-neck PDOX mouse model & peritonitis carcinomatoses PDOX mouse model | TBR > 2, subcutaneous CRC model  Mean fluorescence intensity was higher for UlgG-800F than uFab2-800F or uFab-800F in all PDOX models [119] |
| Nb13-s775z/ Nb15-s775z | S775z | 775 nm – 795 nm | N = 4 x 2 = 8 CRC, GC and PC(-M) & control PDOX mouse model | TBR = 3.35 ± 0.75 in CRC and TBR = 3.41 ± 0.46 in PC (Nb15-s775z) [120] |

**Table 10: Preclinical research TAG-72 targeting tracers**

| **Fluorescent Agent** | **Fluorophore** | **Excision -Emission wavelength** | **Study size - Target organ(s)** | **Study outcome** |
| --- | --- | --- | --- | --- |
| 3E8.scFv.Cys-IR800 | IRDye800CW | 764 nm – 809 nm | N = 4, non-tumour bearing mice for pharmacokinetics  N = 7 (n = 4, CRC PDOX mouse model & n = 3, control group | 1 nmol dose is sufficient to produce high TBR [121] |
| anti-huCC49-IR800 | IRDye800CW | 774 nm – 789 nm | N = 3, subcutaneous CRC mouse model. N = 1, control  N = 7, CRC PDOX mouse model  N = 3, peritoneal metastatic CRC PDOX mousemodel | TBR = 7.39 (50 μg, 72h) (subcutaneous model)  In the orthotopic model, metastases smaller than 1 mm were fluorescently visualized that were invisible with bright light [122] |
| HuCC49-IR800 | IRDye800CW | 774 nm – 789 nm | N = 5, CRC liver metastases PDOX model | Intra-vital imaging demonstrated clear tumour margins with minimal liver fluorescence 48 h after administration of 50 μg huCC49-IR800 with mean TLR=7.53 (SD±2.76)  [123] |

**Table 11: Preclinical research GGT targeting tracers**

| **Fluorescent Agent** | **Fluorophore** | **Excision -Emission wavelength** | **Study size - Target organ(s)** | **Study outcome** |
| --- | --- | --- | --- | --- |
| GGlu-HMRG | HMRG | 710/750 nm –  810 nm | N = 103, human hepatic specimen (N = 50 HCC, N = 8 ICC, N = 45 CRLM) | Hepatocellular carcinoma (N = 50) Sensitivity = 48%  Specificity = 96%  Intrahepatic cholangiocarcinoma (N = 8) Sensitivity = 100%  Specificity = 100%  CRC liver metastases (N = 45) Sensitivity = 87% Specificity = 100% [124] |
| ABTT-Glu |  | 405 nm – 625/680 nm | Human HCC specimen | High signal-to-noise ratio while keeping fluorescence ABTT-Glu showed excellent specificity and sensitivity [125] |
| Poly-g-BAT | CY NH2 | 661 nm – 700/950 nm | CRC PDOX mouse model  human CRC specimens | TNR = 12.3 (after 3 minutes, PDOX mouse model) [126] |
| Cy-(Bio)-GGT | Cyanine | 650 nm – 830 nm | Subcutaenous and Orthotopic liver tumour model | Cy-Bio-GGT in tumour-bearing mice was 9.8-fold higher than that in healthy mice and 2.7-fold higher than Cy-GGT  Systemic and topical administration possible [127] |

**Table 12: Preclinical research OATP targeting tracers**

| **Fluorescent Agent** | **Fluorophore** | **Excision -Emission wavelength** | **Study size - Target organ(s)** | **Study outcome** |
| --- | --- | --- | --- | --- |
| DZ-1 | heptamethine carbocyanine dye | 767 nm – 798 nm | N = 9, subcutaneous HCC model  N = 6, PDOX HCC model & Liver cirrhosis model  Rabbit HCC model  Liver specimens of HCC patients | Six-fold fluorescence intensity increase in HCC as compared to liver cirrhosis  5-20 fold increase in fluorescence intensity in HCC compare to other organs  [128] |
| MHI-148 | heptamethine carbocyanine dye | 760 / 780 nm – 820/860 nm | N = 2 x 5 = 10, subcutaneous HCC mouse model & ICG control  N = 2 x 5 = 10, PDOX HCC model & ICG control | Two-fold higher TBR of MHI-148 compared to ICG  [130] |
| 99mTc-hHEPATO-Cy5 | Cy5.5 | 640 nm – 665 nm | N = 6 , HCC porcine model & N = 2, ICG control porcine model | Liver accumulation and rapid biliary clearance. The effectiveness of bile clearance was best exemplified by the 2-orders-of-magnitude reduction in count rate for the gallbladder (P = 0.008) over time. [128] |

**Table 13: Preclinical research HER 1/2 targeting tracers**

| **Fluorescent Agent** | **Fluorophore** | **Excision-Emission wavelength** | **Studysize**  **Target organ(s)** | **Study outcome** |
| --- | --- | --- | --- | --- |
| slapatinib YQ-H (01–07) | MPA | 780 nm -800 nm | N = 3, orthotopic CRC mouse model  N = 3, PDOX HCC tumour model  N= 3 orthotopic CRC mouse model (blocking assay)N = 3, control group | TBR ~5.6 for HER2 and ~3.9 for HER1; high specificity and tumour accumulation [131] |
| neutravidin-BODIPY-FL | BODIPY-FL | 503 nm – 512 nm | N=5 peritoneal metastasis PDOX mouse model | Activated fluorescence post-pretargeting; strong TBR contrast; 10-fold amplification of the optical fluorescence signal [132] |
| Trastuzumab-IRDye800CW | IRDye800CW | 774 nm – 789 nm | N = 10, orthotopic gastric cancer mouse model | TBR = 4.25 ± 0.67 at 24 h; strong HER2 IHC correlation and tumour targeting [133] |
| trastuzumab-IR700 | IR700 | 789 nm - 814 nm | N = 7, orthotopic gastric cancer mouse model | After NIR‑PIT, fluorescence signals diminished due to cell killing, but there was no difference in tumour response between Tra‑IR700 and Tra‑IR700/IR800 groups [134] |

**Table 14: Preclinical research Lewis antibody targeting tracers**

| **Fluorescent Agent** | **Fluorophore** | **Excision -Emission wavelength** | **Study size - Target organ(s)** | **Study outcome** |
| --- | --- | --- | --- | --- |
| Anti-CA19-9 | AlexaFluor 488 | 496 nm – 519 nm | PDAC orthotopic mouse model | Successful In-vivo binding to tumour tissue. Differentiation between normal and tumour tissue within pancreas 24h after administration. Microscopic foci revealed which were not visible by white light. [135] |
| Anti-CA19-9 | DyLight 650 | 652 nm – 672 nm | N = 4 x 8 = 32, PDAC metastatic PDOX mouse model (bright light, bright light + neoadjuvant therapy, FGS only, FGS + neoadjuvant therapy) | FGS + neoadjuvant therapy eliminated pancreatic cancer metastases in seven out of eight mice. [136] |
| Anti-CA19-9-IRDye800CW | IRDye800CW | 785 nm – 820 nm | N = 22, PDAC orthotopic mouse model + control  N = 3 subcutaneous PDAC mouse model | PEARL imaging: tumour-to-pancreas ratio was 4.51 (±0.74), and the tumour to the liver ratio (TLR) was 3.05 (±0.60) with CA19-9-IRDye800CW, while the TPR was 1.67 (±0.16) and the TLR was 0.95 (±0.05) for the non-specific control IgG–IRDye800CW.  Clinical imaging system: TPR of 2.34 (±0.44) and a TLR of 2.23 (±0.49), compared to 1.11 (±0.13) and 0.69 (±0.07)  [137] |
| ^89^Zr-^ss^dual-5B1 | Fl-DIBO | 363 nm - 469 nm | N = 7, PDAC orthotopic mouse model | Good delineation of metastases & mapping of sentinel lymph nodes via dual-labeled PET/NIRF tracer [138] |
| huA33-Dye800-TCO  ^64^Cu-Tz-SarAr | Dye800 | 745 nm – 820 nm | CRC xenograft mouse model | Decoupling of radionuclide and targeting vector at the same time of injection. PET imaging and NIRF guided excision possible. Possibility to use short -lived radionuclides. [139] |
| CH88.2-800CW /  CH129-800CW | IRDye 800CW | 710/75nm -810/90nm | CRC and pancreatic tumour mouse model | Mean tumour-to-background ratio (TBR) of 2.2 ± 0.3 (Pearl: 3.1 ± 0.8) was observed in the HT-29 tumours and a TBR of 1.8 ± 0.3 (Pearl: 1.9 ± 0.5) was achieved in the moderate expression BxPC-3 model [140] |
|  |  |  | N = 3 x 3, subcutaneous CRC and PDAC mouse model  N = 3 x 3 orthotopic CRC and PDAC mouse models | Bimodal NIRF/PA imaging after 96h  TBR 4.8±1.4 (CH88.2-800CW) and 4.9±0.5 (CH129-800CW) for CRC mouse model  TBR 2.5±0.3 (CH88.2-800CW) and 2.9±0.4 (CH129-800CW) for PDAC mouse model [141] |
