# Supplementary material for: Tumour‐targeted fluorescence‐guided surgery in gastrointestinal cancer: A systematic review of preclinical and clinical research
Source: Clin Transl Med. 2026 Mar 22;16(3):e70615. doi: 10.1002/ctm2.70615 (PMC13093331; doi:10.1002/ctm2.70615)
Supplement: Supplementary file 2 — Supporting Information [file CTM2-16-e70615-s002.docx]

Search strategy – september 2025

Endnote

((exp digestive system tumor/ OR exp Biliary Tract tumor/ OR exp liver tumor/ OR exp peritoneum metastasis/ OR "Digestive System Neoplas*".ti,ab,kf. OR "Neoplasm of the Digestive System".ti,ab,kf. OR "Cancer of Digestive System".ti,ab,kf. OR "Digestive System Cancer*".ti,ab,kf. OR "Cancer of the Digestive System".ti,ab,kf. OR "Cancers of the Digestive System".ti,ab,kf. OR “Digestive Neoplas*”.ti,ab,kf. OR “Digestive Cancer*”.ti,ab,kf. OR ((biliary or bile or gallbladder or gall bladder or gastrointestinal or GI or GI-tract or esophag* or oesophag* or cecal or cecum or caecal or caecum or appendix or appendic* or colon* or colorectal or sigmoid* or rectal* or rectum or anal* or anus or duodenal* or duodenum or ileal* or ileum or jejunal* or jejunum or hepatic or liver or peritoneal) adj3 (cancer* or tumor* or tumour* or neoplas* or carcinom* or metas* or malign*)))

AND (exp Indocyanine Green/ OR exp fluorescence Imaging/ OR exp Multimodal Imaging/ OR exp Fluorescent Dye/ OR exp Near infrared spectroscopy/ OR "Indocyanine*".ti,ab,kf. OR “ICG”.ti,ab,kf. OR "Vofaverdin".ti,ab,kf. OR "Vophaverdin".ti,ab,kf. OR "Wofaverdin".ti,ab,kf. OR "Cardio Green".ti,ab,kf. OR "Cardiogreen".ti,ab,kf. OR "Optical Imag*".ti,ab,kf. OR "Fluorescen*".ti,ab,kf. OR "Autofluorescen*".ti,ab,kf. OR "Multimodal Imag*".ti,ab,kf. OR "Multimodality Imag*".ti,ab,kf. OR image guided.ti,ab,kf. OR "Hybrid Imag*".ti,ab,kf. OR "Fluorescen*".ti,ab,kf. OR "Fluorochrom".ti,ab,kf. OR "Fluorogenic*".ti,ab,kf. OR "Near-Infrared*".ti,ab,kf. OR "Near Infrared*".ti,ab,kf. OR "Near-Infrared*".ti,ab,kf. OR "NIR Spectrosco*".ti,ab,kf. OR "NIR Spectromet*".ti,ab,kf.)

AND (su.fs OR exp computer assisted surgery/ OR exp colorectal surgery/ OR exp Surgical Oncology/ OR exp General Surgery/ OR exp abdominal surgery/ OR Surgical*.ti,ab,kf. OR Operati*.ti,ab,kf. OR Surger*.ti,ab,kf. OR Proctolog*.ti,ab,kf. OR operation*.ti,ab,kf. OR "invasive procedure*".ti,ab,kf. OR "preoperative".ti,ab,kf. OR "intraoperative".ti,ab,kf. OR "peroperative".ti,ab,kf. OR "perioperative".ti,ab,kf.)

AND (exp tumor marker/ OR "Carcinoembryonic Antigen".ti,ab,kf. OR "CEA".ti,ab,kf. OR "Intratumor*".ti,ab,kf. OR "Intra-tumor*".ti,ab,kf. OR "Intratumour*".ti,ab,kf. OR "Intra-tumour*".ti,ab,kf. OR "Specific tumor*".ti,ab,kf. OR ((Guided or spec* or targ*) adj2 (tumor* or tumour*)).ti,ab,kf. OR "Specific tumour*".ti,ab,kf. OR "Guided tumour*".ti,ab,kf. OR "tumour spec*".ti,ab,kf. OR “tumour-spec*”.ti,ab,kf. OR "tumour targ*".ti,ab,kf. OR “tumour-targ*”.ti,ab,kf. OR “identif*”.ti,ab,kf. OR “tumor-to-background”.ti,ab,kf. OR “tumour-to-background”.ti,ab,kf. OR margin*.ti,ab,kf.))

Pubmed

(("Digestive System Neoplasms"[Mesh] OR "Biliary Tract Neoplasms"[Mesh] OR "Gastrointestinal Neoplasms"[Mesh] OR "Liver Neoplasms"[Mesh] OR "Pancreatic Neoplasms"[Mesh] OR "Peritoneal Neoplasms"[Mesh] OR "Digestive System Neoplas*"[tiab] OR "Neoplasm of the Digestive System"[tiab] OR "Cancer of Digestive System"[tiab] OR "Digestive System Cancer*"[tiab] OR "Cancer of the Digestive System"[tiab] OR "Cancers of the Digestive System"[tiab] OR "Biliary Tract Neoplas*"[tiab] OR "Biliary Tract Cancer*"[tiab] OR "Cancer of the Biliary Tract"[tiab] OR "Bile Duct Neoplas*"[tiab] OR "Bile Duct Cancer*"[tiab] OR "Cancer of the Bile Duct"[tiab] OR “Biliary cancer*”[tiab] OR "Gallbladder Neoplas*"[tiab] OR "Cancer of Gallbladder"[tiab] OR "Gallbladder Cancer*"[tiab] OR "Cancer of the Gallbladder"[tiab] OR "Gall Bladder Cancer*"[tiab] OR "Gall Bladder tumor*"[tiab] OR "Gall Bladder tumour*"[tiab] OR “Gallbladder tumor*”[tiab] OR “Gallbladder tumour*”[tiab] OR "Gastrointestinal Neoplas*"[tiab] OR "Cancer of Gastrointestinal Tract"[tiab] OR "Gastrointestinal Tract Cancer*"[tiab] OR "Gastrointestinal Tract tumor*"[tiab] OR "Gastrointestinal Tract tumour*"[tiab] OR "Gastrointestinal Cancer*"[tiab] OR "Cancer of the Gastrointestinal Tract"[tiab] OR “GI tract tumor*”[tiab] OR “GI-tract tumor*”[tiab] OR “GI tract tumour*”[tiab] OR “GI-tract tumour*”[tiab] OR “GI-tract neoplas*”[tiab] OR “GI tract neoplas*”[tiab] OR “GI-tract cancer*”[tiab] OR "Esophageal Neoplas*"[tiab] OR "Oesophageal Neoplas*"[tiab] OR "Cancer of Esophagus"[tiab] OR "Cancer of Oesophagus"[tiab] OR "Esophageal Cancer*"[tiab] OR "Oesophageal Cancer*"[tiab] OR "Cancer of the Esophagus"[tiab] OR "Cancer of the Oesophagus"[tiab] OR "Esophagus Cancer*"[tiab] OR "Esophagus Tumor*"[tiab] OR "Esophagus Tumour*"[tiab] OR "Oesophagus Cancer*"[tiab] OR "Oesophagus Tumor*"[tiab] OR "Oesophagus Tumour*"[tiab] OR "Intestinal Neoplas*"[tiab] OR "Intestines Neoplas*"[tiab] OR "Intestines Cancer*"[tiab] OR "Intestinal Cancer*"[tiab] OR "Cancer of the Intestines"[tiab] OR "Cecal Neoplas*"[tiab] OR "Caecal Tumor*"[tiab] OR "Caecal Tumour*"[tiab] OR "Caecal Neoplas*"[tiab] OR "Cancer of Cecum"[tiab] OR "Cancer of Caecum"[tiab] OR "Cecal Cancer*"[tiab] OR Caecal Cancer*[tiab] OR "Cancer of the Cecum"[tiab] OR "Cancer of the Caecum"[tiab] OR "Appendiceal Neoplas*"[tiab] OR "Appendiceal Cancer*"[tiab] OR "Appendiceal Tumor*"[tiab] OR "Appendiceal Tumour*"[tiab] OR "Appendix Cancer*"[tiab] OR "Cancer of Appendix"[tiab] OR "Colorectal Neoplas*"[tiab] OR "Colorectal Tumour*"[tiab] OR "Colorectal Tumor*"[tiab] OR "Colorectal Cancer*"[tiab] OR "Colorectal Carcinoma*"[tiab] OR "Colonic Neoplas*"[tiab] OR "Colon Neoplas*"[tiab] OR "Cancer of Colon"[tiab] OR "Colon Cancer*"[tiab] OR "Cancer of the Colon"[tiab] OR "Cancers of the Colon"[tiab] OR “Colon Tumor*”[tiab] OR “Colon Tumour*”[tiab] OR "Colonic Cancer*"[tiab] OR "Colon Adenocarcinoma*"[tiab] OR "Sigmoid Neoplas*"[tiab] OR "Sigmoid Colon Neoplas*"[tiab] OR "Sigmoid Cancer*"[tiab] OR "Sigmoid Tumor*"[tiab] OR "Sigmoid Tumour*"[tiab] OR "Cancer of Sigmoid"[tiab] OR "Cancer of the Sigmoid"[tiab] OR "Sigmoid Colon Cancer*"[tiab] OR "Sigmoidal Cancer*"[tiab] OR “Sigmoid cancer*”[tiab] OR "Rectal Neoplas*"[tiab] OR "Rectum Neoplas*"[tiab] OR "Rectal Tumor*"[tiab] OR "Rectal Tumour*"[tiab] OR “rectum Tumor”[tiab] OR “rectum Tumour”[tiab] OR "Cancer of Rectum"[tiab] OR "Rectum Cancer*"[tiab] OR "Cancer of the Rectum"[tiab] OR "Rectal Cancer*"[tiab] OR "Rectum Cancer*"[tiab] OR "Anus Neoplas*"[tiab] OR "Anal Neoplas*"[tiab] OR "Anal Cancer*"[tiab] OR "Anal Tumor*"[tiab] OR "Anal Tumour*"[tiab] OR "Cancer of Anus"[tiab] OR "Anus Cancer*"[tiab] OR "Cancer of the Anus"[tiab] OR "Duodenal Neoplas*"[tiab] OR "Duodenal Cancer*"[tiab] OR “Duodenal Tumor*”[tiab] OR “Duodenal Tumour*”[tiab] OR "Cancer of Duodenum"[tiab] OR "Duodenum Cancer*"[tiab] OR "Cancer of the Duodenum"[tiab] OR "Ileal Neoplas*"[tiab] OR "Cancer of the ILEUM"[tiab] OR "Ileal Cancer*"[tiab] OR "ILEUM Cancer*"[tiab] OR “Ileal tumor*”[tiab] OR “Ileal tumour*”[tiab] OR "Jejunal Neoplas*"[tiab] OR "Cancer of the Jejunum"[tiab] OR "Jejunum Cancer*"[tiab] OR "Jejunal Cancer*"[tiab] OR "Jejunal Tumor*"[tiab] OR "Jejunal Tumour*"[tiab] OR "Hepatic Neoplas*"[tiab] OR “Hepatic tumor*”[tiab] OR “Hepatic tumour*”[tiab] OR "Liver Neoplas*"[tiab] OR "Liver Tumor*"[tiab] OR "Liver Tumour*"[tiab] OR "Cancer of Liver"[tiab] OR "Liver Cancer*"[tiab] OR "Hepatocellular Cancer*"[tiab] OR "Cancer of the Liver"[tiab] OR "Hepatic Cancer*"[tiab] OR "Liver metas*"[tiab] OR "Pancreatic Neoplas*"[tiab] OR "Pancreatic Tumor*"[tiab] OR "Pancreatic Tumour*"[tiab] OR "Pancreas Neoplas*"[tiab] OR "Cancer of Pancreas"[tiab] OR "Pancreas Cancer*"[tiab] OR "Cancer of the Pancreas"[tiab] OR "Pancreatic Cancer*"[tiab] OR "Pancreatic Carcinom*"[tiab] OR "Pancreatic Acinar Carcinom*"[tiab] OR "Peritoneal Neoplas*"[tiab] OR "Peritoneal Cancer*"[tiab] OR "Peritoneal Surface Malignan*"[tiab] OR "Peritoneal Carcinomatos*"[tiab] OR “Peritoneal metas*”[tiab]) AND ("Indocyanine Green"[Mesh] OR "Optical Imaging"[Mesh] OR "Multimodal Imaging"[Mesh] OR "Fluorescent Dyes"[Mesh] OR "Spectroscopy, Near-Infrared"[Mesh] OR "Indocyanine Green*" [tiab] OR “ICG”[tiab] OR "Vofaverdin"[tiab] OR "Vophaverdin"[tiab] OR "Wofaverdin"[tiab] OR "Cardio Green"[tiab] OR "Cardiogreen"[tiab] OR "Optical Imag*"[tiab] OR "Fluorescen*"[tiab] OR "Autofluorescen*"[tiab] OR "Multimodal Imag*"[tiab] OR "Multimodality Imag*"[tiab] OR image guided[tiab] OR "Hybrid Imag*"[tiab] OR "Fluorescen*"[tiab] OR "Fluorochrom"[tiab] OR "Fluorogenic*"[tiab] OR "Near-Infrared*"[tiab] OR "Near Infrared*"[tiab] OR "NIR Spectrosco*"[tiab]) AND (surgery [Subheading] OR "Surgery, Computer-Assisted"[Mesh] OR "Colorectal Surgery"[Mesh] OR "Surgical Oncology"[Mesh] OR "General Surgery"[Mesh] OR "Digestive System Surgical Procedures"[Mesh] OR Surgical*[tiab] OR Operati*[tiab] OR Surger*[tiab] OR Proctolog*[tiab] OR "Operative procedure*"[tiab] OR operation*[tiab] OR "invasive procedure*"[tiab] OR "preoperative"[tiab] OR "intraoperative"[tiab] OR "peroperative"[tiab] OR "perioperative"[tiab]) AND (“Biomarkers, Tumor”[Mesh] OR "Carcinoembryonic Antigen"[tiab] OR "CEA"[tiab] OR "Intratumor*"[tiab] OR "Intra-tumor*"[tiab] OR "Specific tumor*"[tiab] OR "Guided tumor*"[tiab] OR "tumor spec*"[tiab] OR “tumor-spec*”[tiab] OR "tumor targ*"[tiab] OR “tumor-targ*”[tiab] OR "Intratumour*"[tiab] OR "Intra-tumour*"[tiab] OR "Specific tumour*"[tiab] OR "Guided tumour*"[tiab] OR "tumour spec*"[tiab] OR “tumour-spec*”[tiab] OR "tumour targ*"[tiab] OR “tumour-targ*”[tiab] OR “identif*”[tiab] OR margin*[tiab]))
